# Maternal knowledge and attitudes towards complementary feeding in relation to timing of its initiation in rural Bangladesh

**DOI:** 10.1186/s40795-019-0272-0

**Published:** 2019-01-30

**Authors:** Aatekah Owais, Parminder S. Suchdev, Benjamin Schwartz, David G. Kleinbaum, A. S. G. Faruque, Sumon K. Das, Aryeh D. Stein

**Affiliations:** 1 Department of Epidemiology, Rollins School of Public Health and Laney Graduate School, Emory University, Atlanta, GA USA; 2 Hubert Department of Global Health, Rollins School of Public Health, Emory University, 1518 Clifton Road NE. Room 7007, Atlanta, GA 30322 USA; 3 Los Angeles County Department of Public Health, Los Angeles, CA USA; 4 Department of Epidemiology, Emory University, Atlanta, GA USA; 5 International Center for Diarrheal Disease Research, Dhaka, Bangladesh

**Keywords:** Complementary feeding, Timing of initiation, Maternal knowledge and attitudes, Rural Bangladesh

## Abstract

**Background:**

Initiation of complementary feeding is often delayed in Bangladesh and likely contributes to the high burden of infant undernutrition in the country.

**Methods:**

Pregnant women at 28–32 weeks’ gestation were recruited for a cohort-based evaluation of a community-based nutrition education program. To identify predictors of the timing of introduction of solid/semi-solid/soft foods (complementary feeding initiation), we prospectively interviewed 2078women (1042 from intervention area, 1036 from control area) at time of recruitment and at child age 3 and 9 mo. Maternal knowledge and attitudes towards complementary feeding, nutritional importance and cost of complementary foods were assessed at child age 3 months. Two scales were created from the sum of correct responses. Tertiles were created for analysis (Knowledge: 0–7, 8–9, 10–15; Attitudes: 18–25, 26, 27–34). Infant age at complementary feeding initiation was characterized as early (≤4 months), timely (5–6 months) or late (≥7 months), based on maternal recall at child age 9 mo. We used stratified polytomous logistic regression, adjusted for socioeconomic status, infant gender, maternal age, literacy and parity to identify predictors of early or late vs. timely complementary feeding initiation.

**Results:**

Complementary feeding initiation was early for 7%, timely for 49%, and late for 44% of infants. Only 19% of mothers knew the WHO recommended age for complementary feeding initiation. The knowledge score was not associated with timely complementary feeding initiation. Mothers with the most favorable attitudes (highest attitudes score tertile) were more likely to initiate late complementary feeding compared to those with the lowest attitudes score tertile (adjusted OR = 2.2, 95% CI: 1.1–4.4).

**Conclusion:**

Late introduction of complementary foods is still widely prevalent in Bangladesh. Improved maternal knowledge or favorable attitudes towards complementary feeding were not associated with timely introduction of complementary foods, indicating other factors likely determine timing of complementary feeding initiation. This presents an avenue for future research.

## Background

To ensure optimal child growth and development, the World Health Organization (WHO) recommends exclusive breastfeeding for all children up to 6 months of age, followed by introduction of nutritionally adequate and safe complementary foods at 6 months, while continuing breastfeeding until the child is at least 2 years old [1]. However, suboptimal infant and young child feeding (IYCF) practices are prevalent worldwide and are a key determinant of childhood undernutrition, especially between the ages of 3 and 24 months [2]. Specifically, the proportion of infants introduced to complementary foods in a timely manner remains low in many countries. In India and Pakistan, complementary feeding (CF) is initiated for only about half the children aged 6–8 months [3, 4], and in Nigeria, only 64% of children 6–8 months receive complementary foods [5].

Maternal knowledge and attitudes are important determinants of not only child health in general [6–8], but also infant feeding practices in particular [9, 10]. Improving maternal knowledge and attitudes through nutrition counseling and education can lead to improved IYCF practices, and consequently, improved child growth and development, especially in settings with low maternal literacy [11–13].

Bangladesh has a very high burden of childhood undernutrition. The prevalence of children less than 5 years of age with height-for-age, weight-for-age, and weight-for-height z-scores less than − 2.0 is estimated to be 36, 33, and 14%, respectively [14]. Suboptimal infant feeding practices are also widespread. Only 18% of infants 6–11 months receive a minimally acceptable diet, (as defined by WHO [15]) and complementary feeding is delayed for more than one-third of infants aged 6–8 months [14].

Even though delayed initiation of complementary feeding is prevalent in many parts of the world, few studies have tried to identify factors associated with this delay; almost all focus on infant weight/height gain and diet [16]. The few studies that have focused on identifying factors associated with introduction of solid/semi-solid/soft food have used cross-sectional data [9, 17, 18], and are subject to methodological limitations, such as reverse causality. Therefore, well-designed, prospective studies are needed to identify the barriers to timely complementary feeding initiation and isolate those barriers that are modifiable. This study addresses this gap in knowledge by using data collected for the evaluation of a community-based infant nutrition program implemented in rural Bangladesh to assess the association of maternal knowledge of, and attitudes towards complementary feeding with infant age at which complementary foods are introduced.

## Methods

### Study population and setting

Data for this study were collected in the context of an evaluation of a community-based infant and young child nutrition program, called *Akhoni Shomay*. The program was implemented starting in May 2011 in Karimganj, a rural sub-district of Kishoreganj with a population of ~ 320,000, approximately 120 km north of Dhaka. To evaluate the program, a prospective cohort of 2400 pregnant women was recruited, consisting of 1200 women from Karimganj and 1200 from a neighboring sub-district (Katiadi). *Akhoni Shomay* promoted optimal IYCF practices through individual counselling for mothers, as well as group counselling for other key influencers of IYCF practices, such as fathers and grandmothers. The program also encouraged home-based fortification of complementary foods using micronutrient powders.

Women were recruited during their 7th month of gestation, in three waves: January and February 2011, May and June 2011, and September and October 2011. The recruitment waves were designed to coincide with just prior to program launch, immediately after program launch and several months after program launch (to allow for adequate program dissemination), respectively. Follow-up of mother-child dyads occurred at 3, 9, 16 and 24 months of child’s age, using back-translated, pilot-tested questionnaires. Detailed descriptions of the intervention program, study setting, population and data collection tools are also available elsewhere [19–23].

### Variable derivation

#### Complementary feeding knowledge

Complementary feeding knowledge was assessed by using a 19-item instrument administered at the 3-month follow-up (see Additional file 1). Questions were derived from the WHO guiding principles for complementary feeding [24], and assessed maternal knowledge of recommended age of complementary feeding initiation, techniques for responsive feeding, and types of foods and how to prepare them. A scale of complementary feeding knowledge was created from the sum of correct responses. However, if a mother identified force-feeding an infant as acceptable, 1 point was deducted from her knowledge score. The scores were then categorized into tertiles for analysis, with a higher score reflecting better knowledge of optimal IYCF practices.

#### Attitudes

Attitudes were assessed using a 10-item instrument, also administered at the 3-month follow-up, and addressed nutritional importance and cost of complementary foods and nutritional supplements, and ease of continued breastfeeding for the mother. Respondents answered using a 5-point scale ranging from ‘1 = Strongly Agree’ to ‘5 = Strongly Disagree’. Favorable attitudes were reverse-coded before analysis. Factor analysis using principal-axis factoring method and an orthogonal varimax rotation extracted three factors. Eight of the 10 items had factor loadings of > 0.5. An overall attitude scale was created from the simple sum of scores for these eight items (theoretical range: 8–40). The scores were then categorized into tertiles for analysis, with a higher score reflecting more favorable attitudes towards complementary feeding.

#### Infant age at complementary feeding initiation

Infant age (in months) at complementary feeding initiation was estimated using maternal recall at 9-month follow-up (see Additional file 2). Complementary feeding was considered early if reported age was ≤4 months, timely at age 5–6 months, and late if reported age was ≥7 months.

#### Other covariates

Information on household characteristics and ownership of assets, as well as maternal age, literacy and parity was collected at enrollment into the study at 28–32 weeks’ gestation.

### Statistical analysis

Data were imported into Statistical Analysis Software (SAS), version 9.3 for analysis (see Additional file 3). For categorical variables, frequencies and percentages were calculated; for continuous variables, median (range) was calculated. Household socioeconomic status was assessed via characteristics of the respondents’ dwelling and ownership of assets. An asset based socioeconomic status score was created using methods described by Filmer and Pritchett [25].

A 3-level categorical variable was created for infant age at complementary feeding initiation, with timely initiation as referent. Tertiles were created for maternal knowledge and attitude scores for analysis at 0–7, 8–9, and 10–15 for the knowledge score and at 18–25, 26, and 27–34 for the attitudes score.

The study population was stratified into four groups for analysis, based on district of residence and wave of enrollment. All participants from Katiadi, the control district, were included in one group, while participants from Karimganj, the intervention district, were divided into three groups, based on their timing of enrollment into the study and hence potential for exposure to program messaging.

Polytomous logistic regression, stratified by district of residence and wave of enrollment, and adjusted for socioeconomic status, infant gender, maternal age, literacy, and parity, was used to determine the association between maternal knowledge and attitudes and timing of complementary feeding initiation. Using a polytomous regression model, instead of ordinal logistic regression, allows for comparisons of ‘early’ or ‘late’ versus ‘timely’ initiation of complementary feeding, separately. The corresponding odds ratios and their 95% confidence intervals are interpreted using ‘timely’ complementary feeding initiation as referent.

### Ethics

The Research Review Committee (RRC) and the Ethical Review Committee (ERC) of the International Center for Diarrheal Disease Research, Bangladesh (icddr,b) approved the study. Written informed consent was obtained from each woman at the time of enrollment into the study. At each follow-up visit, a description of the information to be collected at that point was provided and verbal consent was obtained.

## Results

The 9-months follow-up was completed by 2078 (86.6%) of the 2400 recruited women. Based on maternal recall, complementary feeding was initiated at age ≤ 4 months for 7%, at 5–6 months for 49%, and ≥ 7 months for 44% of infants. In the intervention district, timely complimentary feeding was initiated for 27.1, 51.0, and 54.7% of infants in the three waves, respectively. In the control district, 53.0% of infants started receiving complimentary foods at 5–6 months of age. By age 9 months, complementary feeding had been initiated for all but three infants.

Table 1 summarizes the maternal knowledge and attitudes regarding complementary feeding prevalent among the mothers in the study, stratified by district of residence and wave of enrollment. Overall, only 17% of mothers answered “6 months” when asked about the recommended age of complementary feeding initiation, with 81% identifying 7 months or older as the right age to introduce complementary foods. When asked about methods of responsive feeding, 26% of respondents in the control district were able to identify 3 or more techniques. In the intervention community the proportion of mothers able to identify 3 or more techniques of responsive feeding increased from 20% among those recruited in the first wave to 61% among those recruited in the third wave. All but 7 of the mothers interviewed “Agreed” or “Strongly Agreed” that complementary foods in addition to breastmilk were healthy for infants > 6 months of age (a favorable attitude), but 97% also “Agreed” or “Strongly Agreed” that feeding their baby food costs more than just breastfeeding (an unfavorable attitude).

**Table 1:** Complementary feeding knowledge and attitudes among 2078 mothers in 2011–2012 in Kishoreganj, Bangladesh, stratified by district of residence and wave of enrollment^a^

Knowledge^b^	%			
	Katiadi^c^(*n* = 1036)	Karimganj, Wave 1 (*n* = 332)	Karimganj, Wave 2 (*n* = 359)	Karimganj, Wave 3 (*n* = 351)
Knew WHO recommended age for complementary feeding initiation is 6 months	28.3	3.9	11.7	2.9
Identified ≥4 food groups for infant’s first foods	92.4	76.8	90.5	91.5
Knew recommended ways to prepare infant’s food	70.9	48.5	88.6	90.6
Identified ≥3 methods of responsive feeding	26.1	19.9	30.9	61.0
Attitudes^b,d^				
Complementary foods in addition to breastmilk are healthy for infants > 6 months	99.4	99.1	99.7	99.4
Nutritional supplements are affordable and ensure infant has adequate nutrition	37.5	17.8	18.7	20.8
Confident about continued breastfeeding	66.6	77.1	82.5	84.1
Complementary feeding is expensive	96.9	96.4	95.0	98.0

^a^ Participants were enrolled in three waves between January and February 2011, May and June 2011, and September and October 2011, respectively

^b^ Assessed at infant age 3 months

^c^ Participants from all three recruitment waves were pooled in the control district

^d^ Proportion includes those who ‘Agreed’ or ‘Strongly Agreed’

Characteristics of study households stratified by tertiles of maternal knowledge and attitudes scores are summarized in Tables 2 and 3, respectively. Maternal age, literacy, parity and socioeconomic status were similar across tertiles of both scores. Timing of complementary feeding initiation differed significantly by tertiles of maternal attitudes (*p* < 0.01) but did not differ across tertiles of knowledge.

**Table 2:** Maternal characteristics^a^ and infant feeding practices^b^ among 2078 mother-child dyads in 2011–2012 in Kishoreganj, Bangladesh, by maternal knowledge

	Maternal knowledge score^c^			
	0–7	8–9	10–15	
*n*	594	699	780	*p*
Maternal characteristics				
Age in years, median (range)	24 (15–49)	24 (15–47)	24 (14–46)	0.18
Literacy, %				0.40
Cannot read at all	36.6	32.9	36.0	
Can read part of a sentence	16.2	16.7	14.1	
Can read a complete sentence	47.2	50.4	49.9	
Parity, %				0.13
1	24.7	28.3	29.5	
≥2	75.3	71.7	70.5	
Socioeconomic status, %				0.07
1st quintile (lowest)	23.4	20.0	16.4	
2nd quintile	20.4	20.0	19.4	
3rd quintile	18.4	18.9	22.0	
4th quintile	19.7	19.3	21.0	
5th quintile (highest)	18.2	21.8	21.2	
Infant feeding practices				
Complementary feeding initiation, %				0.08
Early (≤ 4 months)	8.2	7.3	5.4	
Timely (5–6 months)	46.6	46.9	52.2	
Late (≥ 7 months)	45.2	45.8	42.4	

^a^ Assessed at baseline

^b^ Assessed at infant age 9 months

^c^ Assessed at infant age 3 months; categories are tertiles

**Table 3:** Maternal characteristics^a^ and infant feeding practices^b^ among 2078 mother-child dyads in 2011–2012 in Kishoreganj, Bangladesh, by maternal attitude

	Maternal attitude score^c^			
	18–25	26	27–34	
*n*	646	813	733	*p*
Maternal characteristics				
Age in years, median (range)	24 (15–45)	24 (14–49)	24 (15–46)	0.20
Literacy, %				0.86
Cannot read at all	35.9	34.4	34.9	
Can read part of a sentence	16.3	15.4	14.7	
Can read a complete sentence	47.8	50.2	50.3	
Parity, %				0.83
1	28.5	27.9	27.0	
≥ 2	71.5	72.1	73.0	
Socioeconomic status, %				0.67
1st quintile (lowest)	22.9	19.2	18.8	
2nd quintile	18.4	20.2	21.0	
3rd quintile	19.7	20.8	19.7	
4th quintile	18.6	20.0	20.6	
5th quintile (highest)	20.4	19.8	19.9	
Infant feeding practices				
Complementary feeding initiation, %				< 0.01
Early (≤ 4 months)	11.4	4.9	5.1	
Timely (5–6 months)	52.3	46.2	48.7	
Late (≥ 7 months)	36.3	48.9	46.2	

^a^ Assessed at baseline

^b^ Assessed at infant age 9 months

^c^ Assessed at infant age 3 months; categories are tertiles

The associations between maternal knowledge and attitudes towards complementary feeding and timing of complementary feeding initiation, stratified by district of residence and wave of enrollment are summarized in Table 4. Maternal knowledge was not associated with timing of complementary feeding initiation in any group, but maternal attitudes were associated with timing of complementary feeding initiation in two of the four groups. In the control district, mothers in the middle attitudes tertile were less likely to initiate early (vs. timely) complementary feeding (adjusted OR = 0.4; 95% CI: 0.3–0.9) but more likely to initiate late (vs. timely) complementary feeding (adjusted OR: 1.7; 95% CI: 1.2–2.3) compared to mothers in the lowest attitudes tertiles.

**Table 4:** Association between maternal knowledge and attitudes and complementary feeding initiation^a^ in 2011^g^-2012 in Kishoreganj, Bangladesh

	Katiadi (n = 1036)			Karimganj, Wave 1 (n = 332)			Karimganj, Wave 2 (n = 359)			Karimganj, Wave 3 (n = 351)		
	Maternal knowledge score^b^			Maternal knowledge score^b^			Maternal knowledge score^b^			Maternal knowledge score^b^		
	0–7	8–9	10–15	0–7	8–9	10–15	0–7	8–9	10–15	0–7	8–9	10–15
CF initiation												
Unadjusted OR (95% CI)												
Early^c^ vs. timely^d^	Ref	1.06 (0.60–1.87)	0.73 (0.40–1.31)	Ref.	1.05 (0.38–2.88)	1.66 (0.45–6.14)	Ref.	0.75 (0.26–2.16)	0.26 (0.07–1.00)	Ref.	1.57 (0.17–14.8)	1.79 (0.21–15.0)
Late^e^ vs. timely^d^	Ref	1.14 (0.81–1.60)	1.13 (0.81–1.57)	Ref.	1.29 (0.74–2.27)	2.30 (1.04–5.06)	Ref.	1.51 (0.89–2.57)	0.78 (0.45–1.34)	Ref.	1.01 (0.49–2.07)	1.00 (0.51–1.96)
Adjusted^f^ OR (95% CI)												
Early^c^ vs. timely^d^	Ref	1.12 (0.63–1.98)	0.74 (0.41–1.34)	Ref.	1.14 (0.40–3.23)	1.82 (0.48–6.93)	Ref.	0.73 (0.24–2.20)	0.30 (0.08–1.22)	Ref.	1.51 (0.15–15.39)	1.92 (0.21–17.35)
Late^e^ vs. timely^d^	Ref	1.14 (0.81–1.61)	1.15 (0.82–1.60)	Ref.	1.39 (0.78–2.47)	2.50 (1.12–5.59)	Ref.	1.54 (0.90–2.64)	0.82 (0.47–1.43)	Ref.	0.30 (0.08–1.22)	1.17 (0.57–2.39)
	Maternal attitude score^b^			Maternal attitude score^b^			Maternal attitude score^b^			Maternal attitude score^b^		
	18–25	26	27–34	18–25	26	27–34	18–25	26	27–34	18–25	26	27–34
CF initiation												
Unadjusted OR (95% CI)												
Early^c^ vs. timely^d^	Ref	0.49 (0.28–0.86)	0.66 (0.38–1.17)	Ref.	0.26 (0.09–0.77)	0.45 (0.15–1.35)	Ref.	0.88 (0.29–2.62)	0.49 (0.13–1.85)	Ref.	0.58 (0.12–2.76)	0.45 (0.12–1.68)
Late^e^ vs. timely^d^	Ref	1.65 (1.22–2.23)	1.10 (0.78–1.56)	Ref.	1.29 (0.70–2.40)	2.11 (1.08–4.12)	Ref.	1.62 (0.91–2.89)	1.67 (0.92–3.05)	Ref.	0.83 (0.42–1.65)	0.97 (0.55–1.73)
Adjusted^f^ OR (95% CI)												
Early^c^ vs. timely^d^	Ref	0.41 (0.28–0.86)	0.68 (0.38–1.21)	Ref.	0.25 (0.08–0.77)	0.41 (0.13–1.29)	Ref.	0.74 (0.24–2.35)	0.49 (0.13–1.89)	Ref.	0.59 (0.11–3.10)	0.41 (0.10–1.62)
Late^e^ vs. timely^d^	Ref	1.67 (1.24–2.26)	1.14 (0.80–1.61)	Ref.	1.37 (0.73–2.59)	2.21 (1.11–4.40)	Ref.	1.62 (0.90–2.92)	1.63 (0.89–2.99)	Ref.	0.86 (0.42–1.74)	0.92 (0.51–1.65)

^a^ Assessed at infant age 9 months

^b^ Assessed at infant age 3 months; categories are tertiles

^c^ Defined as age ≤ 4 months

^d^ Defined at age 5–6 months

^e^ Defined as age ≥ 7 months

^f^ Adjusted for SES, maternal age, literacy, parity, and infant gender

^g^ Participants were enrolled in three waves between January and February 2011, May and June 2011, and September and October 2011, respectively

In Karimganj, the intervention district, maternal attitudes towards complementary were significantly associated with timing of complementary feeding initiation only for those enrolled in the first wave. Mothers in the middle attitudes tertile were less likely to initiate early (vs. timely) complementary feeding compared to mothers in the lowest attitudes tertile (adjusted OR = 0.3; 95% CI: 0.1–0.8). Mothers in the highest attitudes tertile were more likely to initiate late (vs. timely) complementary feeding compared to mothers in the lowest attitudes tertile (adjusted OR = 2.2; 95% CI: 1.1–4.4). Household socioeconomic status, maternal age, literacy, parity and infant gender were not associated with timing of complementary feeding initiation.

## Discussion

This study used prospectively collected data to assess the association between maternal knowledge and attitudes regarding complementary feeding and the timing of complementary feeding initiation in rural Bangladesh. We observed that although the prevalence of early initiation is low, a large proportion of mothers are waiting longer than the WHO recommended age of 6 months before introducing solid/semi-solid/soft foods to infants, and that more favorable attitudes were associated with late initiation of complementary feeding.

Delayed introduction to complementary foods is not unique to this study setting. In several countries with high burden of childhood undernutrition, complementary feeding is initiated later than recommended for a significant proportion of infants [14, 26, 27]. Given this fact, as well as the well-established relationship between suboptimal complementary feeding practices and consequences for child health and survival, few nutrition intervention programs have focused on how maternal attitudes can affect timing of complementary feeding initiation [12, 13]. To our knowledge, this is the first study to do so.

We found that maternal attitudes towards, but not maternal knowledge about complementary feeding, were associated with the timing of complementary feeding initiation. Specifically, when comparing infants for whom complementary feeding was initiated early or on time, mothers with more favorable attitudes towards complementary feeding were less likely to introduce foods to their infants early. However, when comparing infants for whom complementary feeding was initiated too late or on time, mothers with better attitudes were more likely to initiate late complementary feeding. We believe this is an important finding and presents an avenue for future research.

Neither maternal education as measured by literacy status nor context-specific knowledge regarding IYCF practices was associated with timing of introduction of solid/semi-solid/soft foods in this setting. This finding is in contrast to that of other studies that have assessed factors influencing timing of complementary feeding initiation [17, 18, 28]. One reason for this null finding may be that maternal knowledge/education may not be the foremost driver of child rearing practices in this particular setting. Working in the same setting, Yu et al. [20] did not observe an association between prenatal maternal breastfeeding knowledge and exclusive breastfeeding status at infant age 3 months, and Owais et al. [21] did not find an association between maternal literacy and receipt of minimally acceptable diet (as defined by WHO [15]) at infant age 9 months.

This study uses data from a cohort-based evaluation of a community-based nutrition intervention program. The intervention program was successful in improving IYCF practices of timely complimentary feeding initiation, as well as the quality of infant diet, assessed at 9 months [22].

A major strength of this study is the large sample size, which increases the power of analysis. Furthermore, maternal knowledge and attitudes were measured at infant age 3 months, prior to when infant age at complementary feeding initiation was assessed. This strengthens the ability to make causal inferences. However, as with any other observational study, this one also has some limitations. The outcome is based on maternal recall up to several months after solid/semi-solid/soft foods were introduced to the infant. This is a potential source of outcome misclassification in the study as infant age at complementary feeding initiation was assigned based on maternal report and not on directly observed feeding practices. The time lag may also be a potential source of outcome misclassification as maternal recall may have been affected. However, we did cross-reference the reported age of complementary feeding initiation at the 9-month follow-up with infant diet (based on 24-h maternal recall) reported at the 3-month follow-up. A discrepancy was present for only 3% of infants included in this study and adjustments to the age at complementary feeding initiation were made accordingly.

## Conclusion

In conclusion, we observed that the proportion of infants receiving complementary foods too early was low and late introduction of foods remains widely prevalent. These findings imply that interventions, such as educational activities, aimed at improving infant nutritional status need to focus on emphasizing timely complementary feeding initiation. However, identifying barriers to optimal feeding practices should remain a research priority. A better understanding of predictors of behaviors around infant feeding will enable interventions programs to be more effective in modifying these behaviors and lead to improved child growth and development.

## Additional files

Additional file 1: Questionnaire used at child age 3 mo. (PDF 782 kb)

Additional file 2: Questionanaire used at child age 9 mo. (PDF 1192 kb)

Additional file 3: Analytic data set. (XLS 242 kb)

## Abbreviations

CF: Complementary feeding
ERC: Ethical Review Committee
icddr, b: International Center for Diarrheal Disease Research, Bangladesh
IYCF: Infant and young child feeding
OR: Odds ratio
RRC: Research Review Committee
WHO: World Health Organization
