# Supplementary material for: Maternal knowledge and attitudes towards complementary feeding in relation to timing of its initiation in rural Bangladesh
Source: BMC Nutr. 2019 Jan 30;5:7. doi: 10.1186/s40795-019-0272-0 (PMC7050709; doi:10.1186/s40795-019-0272-0)
Supplement: Supplementary file 1 — Questionnaire used at child age 3 mo. (PDF 782 kb) [file 40795_2019_272_MOESM1_ESM.pdf]

Study ID | | | | |

Monitoring growth and nutritional status of children in rural Bangladesh
Three Month Household Questionnaire

1. Identification

Read: To begin with, I would like to collect some basic identification information from you.

	Date of Interview:	__ __ / __ __ / __ __ __ __
	Mobile no:	Own<br>Request<br>Name:
1.1	Child's mother name:______________________	Mother Study ID:
1.2	(MOTHER NAME), in what month and year were you born ***(month and year)***	month<br>88= Don't Know Month<br>year<br>8888= Don't Know Year
1.3a	How old were you at your last birthday? ***(compare with question 1.2 above for consistency)***	Age in completed years ------------
1.3b	Do you have a government ID card where your birth date is written down?<br><br>IF YES:<br>May I see it? Verify age and return to respondent.<br><br>01 = Yes<br>02 = No<br>03 = No card<br>88 = Don't Know	
1.4	Child's father name :______________________	
1.5	Name of Head of Household:______________________	
1.6	Area:	01= Karimganj<br>02= Katiadi ,
1.7	Union ,Ward:	
1.8	Village/Block/Mohalla:	

Study ID

1.9	***Is (INFANT NAME) alive today?***	01 = Yes<br>02 = No >> ***Skip to "Verbal Autopsy Supplement" (Do NOT need to complete rest of this questionnaire)***<br><br>03 = Declined to answer<br>88 = Don't know	

1.10	In the next 6 months are you planning to live at another place, different from the place you are living now?	01 = Yes<br>02 = No >> ***Skip to 1.12***<br>88 = Don't know	
1.11	How can we best reach you in 6 months?<br><br>Instructions: Write down new address, or probe for phone number that will not change in 6 months.		
1.12	Are you currently attending school?	01 = Yes<br>02 = No	
1.13	What is the last class (grade) of school that you have completed?	***Instructions: Insert exact number of years completed.***<br><br>01 = 1 year 08 = 8 years<br>02 = 2 years 09 = 9 years<br>03 = 3 years 10 = 10 years<br>04 = 4 years 11 = 11 years<br>05 = 5 years 12 = 12 years<br>06 = 6 years<br>07 = 7 years<br>13 = Got admitted into the college but did not attend classes<br>14 = Got admitted into the college, attended classes, but did not appear at the exam<br>15 = College and post college degree<br>16 = Some college (undergraduate)<br>20=Got admitted into the school, but never attended any class<br>21= Got admitted into the school, attended classes, but did not appear at the exam<br>33= Can not sign<br>44 = Can sign only<br>55=Religious education only<br>66 = Never went to school<br>88 = Don't Know<br>99 = Not applicable	
1.14	Are you currently working for income?	01 = Yes<br>02 = No<br>99 = Not Applicable	

1.15	What type of work are you doing?	*Instructions: Code for different types of occupations below.*	

Code for 1.15 -Types of Occupations (Circle the occupation)

01=Professional/technical (Doctor, engineer, lawyer, teacher, economist, agriculturist), 02=Large business (≥10,000/ Taka invested), 03=Small business (<10,000/ Taka invested), street vendors, 04=Blue collar services:(Factory worker, industry worker, garment worker), 05=White collar services: (Officer, manager, administrator, clerk), 06=Skilled worker (Driver, potter, black smith, gold smith, carpenter, mason, plumber, mechanic, tailor, handicraft), 07=Un-skilled worker (Boatman, fisherman), 08=Day laborer (Rickshaw/cart puller, construction worker, daily wage labor), 09=Farmer/share cropper, 10=Domestic maid/house maid, 11=House wife, 12=Overseas employment, 13=Beggar, 14=Unemployed, 15=Student, 16=Old aged/inactive, 17=Household work, 99=Not applicable, 77=Other (specify)____________________

Study ID | | | | |

2. Changes in Demographics and Socio-economic Status

Read: Several months ago, we asked about the people usually living in your household. Now I would like to ask you about whether there have been any changes in the number of people living in your household, including any deaths and guests who stayed here last night.

2.1	***Does (FAMILY MEMBER NAME) still usually live in your household? Please include any guests who stayed here last night. Also be sure to tell me if you are the person, or if the person has died.***<br><br>01 = Yes<br>02 = No<br>03 = No, person has died<br>04 = Yes, that person is the woman herself.<br>88 = Don't Know<br><br>***Instructions: BEFORE THE SURVEY, please fill out all members of the household listed in the baseline questionnaire (Section 2.1). During the survey, read out each member listed in the baseline questionnaire and record who is still there or who is no longer part of the household.***<br><br>***When the name read out is the woman herself, please enter '04' as the code.***<br><br>***Read: Please be sure to tell me if the name I read is your name.***	Line No.	Family Member Name	
		01		|__|__|
		02		|__|__|
		03		|__|__|
		04		|__|__|
		05		|__|__|
		06		|__|__|
		07		|__|__|
		08		|__|__|
		09		|__|__|
		10		|__|__|
		11		|__|__|
		12		|__|__|
		13		|__|__|
		14		|__|__|

Study ID

2.2 Household Schedule

Read: We would like to now include any additional people in your household who were not previously recorded. First, we would like to start with your newborn infant (or infants if you had multiple births at the same time).

Line no.	Usual resident and visitors	Relationship to head of household	Sex	Residence		Age	Marital Status	Ever attended school	Level of school attended	Current school attendance	Current paid work status	Current type of work
1	2	3	4	5		6	7	8	9	10	11	12
-------------------------------- List the names of your newborn infant(s) and record the sex as well as other information.		What is the relationship of (NAME) to the head of the household? ---------- *See codes below. Include Relationship Code*	Is (NAME) male or female? ------- 1=M 2=F	Does (NAME) usually live here? ------- 1=Yes 2=No	Did (NAME) stay here last night? ------- 1=Yes 2=No	How old is (NAME)? ------- (complete year) *If age is less than 1 year write '00'* 88=Don't Know	What is (NAME) current marital status? ------------ 01=currently married 02=divorced /separated/ deserted/ widowed 03=never-married 04=Widower 88=Don't Know, 99 = Not Applicable	Has (NAME) ever attended school? --------- 01=Yes 02=No 88=Don't Know, 99=Not Applicable	What is the level of school (NAME) has last attended? ----------- *Insert exact number of years completed* 33=Can't signature, 44=Can signature only, 55=Religious education only 66=Never went to school 88=Don't know 99=Not Applicable	Is (NAME) currently attending school? -------- 01=Yes 02=No 88=Don't Know, 99=Not Applicable	Is (NAME) currently working? --------------- 01=Yes 02=No 88=Don't Know, 99=Not Applicable no need	What type o work is (NAME) currently doing? ------- Use codes given below 99= Not Applicable (in case of a child)
01							9 9	9 9	9 9	9 9	9 9	9 9
02							9 9	9 9	9 9	9 9	9 9	9 9
03							9 9	9 9	9 9	9 9	9 9	9 9

Study ID [ ][ ][ ][ ]

Line no.	Usual resident and visitors	Relationship to head of household	Sex	Residence		Age	Marital Status	Ever attended school	Level of school attended	Current school attendance	Current paid work status	Current typ[e] of work
1	2	3	4	5		6	7	8	9	10	11	12
Read: We would like to now include any additional people in your household who were not previously recorded. Please give me the names of <u>only any additional</u> people who now usually live in your household including any guests who stayed here last night.<br><br>***Instructions: Please start with newborn infant(s) first, and record on the previous page.***<br><br>--------------------------------<br><br>List the names and record the relationship and sex for each person.		What is the relationship of (NAME) to the head of the household?<br><br>----------<br>*See codes below. Include Relationship Code*	Is (NAME) male or female?<br><br>-------<br><br>1=M, 2=F	Does (NAME) usually live here?<br><br>-------<br><br>1=Yes<br>2=No	Did (NAME) stay here last night?<br><br>-------<br><br>1=Yes<br>2=No	How old is (NAME)?<br><br>-------<br><br>(complete year) *If age is less than 1 year write '00'*<br><br>88=Don't Know	What is (NAME) current marital status?<br><br>------------<br>01=currently married<br>02=divorced /separated/ deserted/ widowed<br>03=never-married<br>04=Widower<br>88=Don't Know,	Has (NAME) ever attended school?<br><br>---------<br><br>01=Yes<br>02=No<br>88=Don't Know, `	What is the level of school (NAME) has last attended?<br><br>-----------<br>*Insert exact number of years completed*<br>33=Can't signature,<br>44=Can signature only,<br>55=Religious education only<br>66=Never went to school<br>88=Don't know<br>99=Not Applicable	Is (NAME) currently attending school?<br><br>--------<br><br>01=Yes<br>02=No<br>88=Don't Know,<br>99=Not Applicable	Is (NAME) currently working?<br><br>--------------<br><br>01=Yes<br>02=No<br>88=Don't Know,<br>99=Not Applicable no need	What type o[f] work is (NAME) currently doing?<br><br>-------<br>Use codes given below<br>99= Not Applicable (in case of a child)
01		[ ][ ]	[ ]	[ ]	[ ]	[ ][ ]	[ ][ ]	[ ][ ]	[ ][ ]	[ ][ ]	[ ][ ]	[ ][ ]
02		[ ][ ]	[ ]	[ ]	[ ]	[ ][ ]	[ ][ ]	[ ][ ]	[ ][ ]	[ ][ ]	[ ][ ]	[ ][ ]
03		[ ][ ]	[ ]	[ ]	[ ]	[ ][ ]	[ ][ ]	[ ][ ]	[ ][ ]	[ ][ ]	[ ][ ]	[ ][ ]

Study ID

04													
05													
06													
07													
08													
09													
10													

Codes for question no. 3: Relationship with the household head
01 = Household head, 02 = Spouse, 03 = Daughter/son, 04 =Son-in-law/daughter-in-law, 05 =Grand son/grand daughter, 06 =Father/mother, 07 = Father-in-law/mother-in-law
08 = Brother-in-law/sister-in-law, 09 = Other relative(Specify)-----------------------, 10= Foster child/step child, 11= Brother/sister, 12= Non-relative, 88=Don't know
77= Others(Specify)---------------------------------

Study ID | | | | |

<u>Codes of occupation for question no. 12</u>

01=Professional/technical (Doctor, engineer, lawyer, teacher, economist, agriculturist), 02=Large business (≥10,000/ Taka invested), 03=Small business (<10,000/ Taka invested), street vendors, 04=Blue collar services: (Factory worker, industry worker, garment worker), 05=White collar services: (Officer, manager, administrator, clerk), 06=Skilled worker (Driver, potter, black smith, gold smith, carpenter, mason, plumber, mechanic, tailor, handicraft), 07=Un-skilled worker (Boatman, fisherman), 08=Day labor (Rickshaw/cart puller, construction worker, daily wage labor), 09=Farmer/share cropper, 10=Domestic maid/house maid, 11=House wife, 12=Overseas employment, 13=Beggar, 14=Unemployed, 15= Student, 16= Aged, 17= Household task, 99=Not Applicable, 88= Don't know,77=Other (specify)___________________

2.3	***Read: Now, I would like to make sure that we have included everyone in our list of people in your household.***<br><br>***Have we included your newborn infant(s)?***	01 = Yes<br>02 = No<br><br>88 = Don't Know	|_|_|
2.4	***Have we now included all other people in your household in our listing?***<br><br>***Instructions: If "No", complete detailed Household Schedule Information below.***	01 = Yes<br>02 = No<br><br>88= Don't Know	|_|_|

2.5	***(MOTHER NAME), what is your current marital status?***	01=Married, 1st wife<br>02 = Married, 2nd wife<br>03 = Married, 3rd wife<br>04 = Married, 4th wife<br><br>05 = Single, never married<br>06 = Single, separated<br>07= Single, widowed<br>08= Single, divorced<br>09 = Deserted<br>88 = Declined to answer<br>99=Not Applicable<br>77 = Other. Please Specify ______	|_|_|

Study ID | | | | |

3. Food Securities and Maternal Diet and Nutrition

Read: Now I would like to ask you some questions about your family's habits relating to food.

Q-01.	During the last 30 d, at what interval has your household purchased rice?	1. Did not buy<br>2. 1–3 times the last 30 d<br>3. Once in 7 d<br>4. 2–3 times in 7 d<br>5. At least 4–5 times in 7 d	☐
Q-02.	During the last 30 d, at what interval has your household purchased "kanchabajar"? (Note: "kanchabajar" refers to shopping of perishable food items such as vegetables, fish and meat.)	1. Did not buy<br>2. 1–3 times the last 30 d<br>3. Once in 7 d<br>4. 2–3 times in 7 d<br>5. At least 4–5 times in 7 d	☐
Q-03.	During the last 30 d, how many times a day did cooking usually take place in your household?	1. Never<br>2. Once a day<br>3. Twice a day<br>4. Three times a day<br>5. Four times or more	☐
Q-04.	During the last 30 d, has your household helped others with cash or food items (like rice) for enabling them to make a meal? (If the girl/woman is poor, tell her that we need to ask this question of everybody, so she does not mind.)	1 = Yes<br>2 = No	☐
Q-05.	During the last 30 d, how often has your household had to borrow from others to make a meal? (If the woman is rich, tell her that we need to ask this question of everybody, so she does not mind.)	1. Never >> Skip to Q-07.<br>2. 1–3 times in the last 30 d<br>3. Once in 7 d<br>4. 2–3 times in 7 d<br>5. At least 4–5 times in 7 d	☐
Q-06.	Have you paid back or do you think you can pay back?	1 = Yes<br>2 = No	☐
Q-07.	During the last 30 d, how many times on average have you had a fulfilling meal in a day?	1. One time<br>2. Two times<br>3. Three times<br>4. Four times	☐
Q-08.	During the last 30 d, how often has it happened that you could NOT eat as many fulfilling meals as you would like to have done?	1. Never<br>2. Less than once in 7 d<br>3. Once in 7 d<br>4. 2–3 times in 7 d<br>5. At least 4–5 times in 7 d	☐
Q-09.	For the last 30 d, did you usually have snacks in between meals?	1.No<br>2. Once or twice<br>3. Three times or more	☐
Q-10.	For the last 30 d, how often did you have fish/meat?<br>(If the girl/woman is rich, tell her that we need to ask this question of everybody, so she does not mind.)	1.Not once<br>2.Less than once in 7 d<br>3.Once in 7 d<br>4.2–3 times in 7 d<br>5.At least 4–5 times in 7 d	☐
Q-11.	During the last 30 d, how often have you had to eat rice with just chili and salt?	1.Did not have to<br>2.Less than once in 7 d<br>3.Once in 7 d<br>4.2–3 times in 7 d<br>5.At least 4–5 times in 7 d	☐

Food Groups and Weights

Read: Now I would like to ask you some questions regarding your diet and nutrition. I would like to record any liquids or foods that you had in the past 7 days.

3.1	Food items	Food groups	Weight	Number of days consumed (in last 7 days)
A.	Maize, Rice, porridge, smashed rice/rice gruel, bread and other cereals	Cereals and Tubers	**2**	
B.	Cassava, potatoes and sweet potatoes			
C.	Beans, peas, groundnuts and cashew nut	Pulses	**3**	
D.	Vegetables and leaves	Vegetables	**1**	
E.	Fruits	Fruit	**1**	
F.	Beef, goat, poultry, pork, eggs, dried fish and fish	Meat and fish	**4**	
G.	Milk, yogurt and other dairy	Milk	**4**	
H.	Sugar and sugar products	Sugar	**0.5**	
I.	Oils, fats and butter	Oil	**0.5**	
J.	Condiments	Condiments	**0**	

Dietary Diversity: Individual Dietary Diversity Questionnaire (IDDS)

Read: Now I would like to ask you some questions regarding your diet and nutrition. I would like to record any liquids or foods that you had in the past 24 hours.

Instructions: Please code '1' if the mother has consumed the food mentioned in the list in past 24 hours, otherwise code '2 Please circle the food name that has been consumed.

3.2	*Food list*	Example	*1=Yes, 2=No*
A.	CEREALS	Rice, smashed rice/rice gruel, bread, noodles, biscuits or any other foods made from wheat or rice	
B.	VITAMIN A RICH VEGETABLES AND TUBERS	pumpkin, carrots, sweet potatoes and other locally available vitamin-A rich vegetables	
C.	WHITE TUBERS AND ROOTS	White potatoes or foods made from roots	
D.	DARK GREEN LEAFY VEGETABLES	dark green/leafy vegetables locally available vitamin-A rich leaves (e.g.amaranth leaves)	
E.	OTHER VEGETABLES	other vegetables (e.g. tomato, eggplant)	
F.	VITAMIN A RICH FRUITS	fruits rich in vitamin A (e.g. ripe mangoes, papaya, jackfruit)	
G.	OTHER FRUITS	other fruits including guava, pineapple, watermelon, melon, orange, apple, grape, banana	
H.	ORGAN MEAT (IRONRICH)	liver, kidney, heart or other organ meats	

I.	FLESH MEATS	beef, lamb, goat, chicken, duck, or other birds	
J.	EGGS	egg	
K.	FISH	fresh or dried fish or shellfish	
L.	LEGUMES, NUTS AND SEEDS	beans, peas, lentils, nuts, seeds or foods made from these	
M.	MILK AND MILK PRODUCTS	Milk (cow's, goat's), cheese, yogurt or other milk products	
N.	OILS AND FATS	Oil, fats or butter added to food or used for cooking	
O.	JUNK FOOD	Cookies (cake, biscuit, cheaps), sweets, samocha	

3.3	***Are you currently taking any iron tablets or iron syrup?***	01 = Yes<br>02 = No<br>88 = Don't know	
3.4	***Did you take any iron tablets or iron syrup during your pregnancy?***	01 = Yes<br>02 = No>>skip to Q-4.1<br>88 = Don't know>>skip to Q-4.1	
3.5	***During this pregnancy, how many months/days did you take iron tablets or syrup?***	Months<br>Days<br>00= None received<br>99 = Not Applicable	
3.6	***During this pregnancy, how many days per week did you take iron tablets or syrup?***	-------------------- Times<br>99 = Not Applicable	
3.7	***During this pregnancy, how many times per day did you take iron tablets or syrup?***	---------------------- number<br>99 = Not Applicable	
3.8	***From where did you receive iron tablets?***	01=Government Hospital<br>02=Upazila Health Complex<br>03=Maternal and Child Welfare Center<br>04=NGO Static Clinic<br>05= FWC<br>06=Private Hospital/Clinic<br>07=Other Private Medical Center<br>08= Pharmacy	

		09 = Village Doctor<br>10= Community Clinic<br>11= Health Worker<br>77= Others(Specify)------------<br>88 = Don't know<br>99 = Not applicable ***(if Q-3.8 'no/don't')***	
3.9	***Did you take all iron tablets that you received?***	01= Yes<br>2= No>>***skip to Q-3.11***<br>88 = Don't know>>***skip to Q-3.11***<br>99 = Not applicable ***(if Q-3.8 'no/don't')***	
3.10	***Why did you take all iron tablets?***	01 = Felt necessary<br>02= Encouraged by family members<br>03= Doctor adviced<br>77= Others(Specify)-------------------------------<br>88 = Don't Know<br>99 = Not applicable ***(if Q-3.8 'no/don't')***	
3.11	***Why did you not take all iron tablets?***	01 = Disliked taste<br>02 = Forgot<br>03 = Disliked side effects<br>04 = Felt unnecessary<br>05 = Gave away iron tablets<br>06= Objection from family<br>07 = Lost of tablet<br>08= Insufficient iron tablet supply<br>09= Unable purchased<br>10= Vertigo<br>77= Others(Specify)-------------------------------<br>88 = Don't Know<br>99 = Not applicable ***(if Q-3.8 'no/don't') (if Q-3.9 'yes')***	

Study ID

4. Delivery

Read: Now I would like to ask you some questions regarding your recent experience in giving birth to your infant.

4.1	What is the name of your **newborn** baby?	______________________	
4.2	Is (INFANT NAME) a boy or a girl?	01 = Boy<br>02 = Girl	
4.3	What date did you give birth to (INFANT NAME)?	Day<br>Month<br>Year	
4.4	How old is (INFANT NAME) today?	Months ______________<br>Days ______________	
4.5	Now we would like to just confirm the birth date of (INFANT NAME).<br><br>Do you have a growth monitoring card where (INFANT NAME)'s vaccinations or identify where this information is written down?<br><br>**IF YES, ask: May I see it please?**	01 = Yes, seen the card<br>02 = Yes, not seen the card<br>03 = No card<br>04=Vaccine is not given yet	
4.6	On the growth monitoring card, what was the age when (INFANT NAME) was first weighed?<br><br>***Instructions: If the child has no growth monitoring card but mother can tell the weight of the child please confirm that Q-4.5 is marked as "03."***	------------------Months<br><br>01= No age of first weight measurement recorded.<br>02 = child has no growth monitoring card>>skip to Q-4.8<br>88 = Don't know<br>99 = Not applicable	
4.7	On the growth monitoring card, what is the total number of times that (INFANT NAME) was weighed?	Number of times-----------------------<br><br>01= No weight recorded.<br>88 = Don't know<br>99 = Not applicable	
4.8	Were there any complications during pregnancy?	01 = Yes<br>02 = No >> Skip to 4.10<br>88 = Don't know>> Skip to 4.10	

4.9	What complications occurred during pregnancy?<br><br>01= Yes<br>02= No<br>88 = Don't Know<br>99 = Not applicable ***(if no response)***	Early pregnancy bleeding<br>Incompetent cervix<br>Diabetes<br>Placenta abruption<br>High blood pressure<br>Rh Disease<br>Group B strep<br>High Fever (fever with chills or rigors)<br>Convulsion<br>Swelling of the limbs<br><br>Other. Specify: ______________________	
4.10	During this pregnancy, were you given an injection in the arm to prevent the baby from getting tetanus, that is, convulsions after birth?	01= Yes<br>02= No >> Skip to 4.12<br>88= Don't Know >> Skip to 4.12	
4.11	How many times did you get this tetanus toxoid injection?	-------------------- times<br>88= Don't Know<br>99= Not applicable	
4.12	Were you aware of any complications that occurred during delivery?	01= Yes<br>02= No	
4.13	Around the time of the birth of (INFANT NAME), did you have any problems?	01= Yes,<br>02= No>> Skip to 4.15	

4.13	Around the time of the birth of (INFANT NAME), did you have any problems?	01= Yes,<br>02= No>> Skip to 4.15<br>88= Don't Know >> Skip to 4.15	
4.14	What were the problems?<br><br>01= Yes<br>02= No<br>88 = Don't Know	Long labor that is regular contractions, which lasted more than 12 hours?	
		Excessive bleeding that was so much that you feared it was life threatening?	
		A high fever with bad smelling vaginal discharge?	
		Convulsions?	
		Baby's hands or feet came first during delivery?	
		Rupture membrane	
		When water broke, hours	
		Days	
		Other. Specify:-------------------------	
4.15	Where did you give birth to (INFANT NAME)?<br><br>INSTRUCTIONS: Probe to identify the type of source and circle the appropriate code.	11=Your Home<br>12=Maternal Family Home<br>21=Government Hospital<br>22=Upazila Health Complex<br>23=Maternal and Child Welfare Center<br>31=NGO Static Clinic<br>41=Private Hospital/Clinic<br>42=Other Private Medical Center<br>77=Others (Specify)---------------------	
4.16	Was (INFANT NAME) born by cesarean section?	01 = Yes<br>02 = No<br>88 = Don't Know	
4.17	After (INFANT NAME) was born, did any medical persons check on your baby's health?	01 = Yes<br>02 = No >> ***Skip to 5.1***<br>88 = Don't Know >> ***Skip to 5.1***	
4.18	How long after delivery did the first check take place?<br><br>***(soon after birth to one month)***	Hours<br>Days	

4.18	How long after delivery did the first check	Hours<br>Days<br>Weeks<br>88=Don't Know<br>99=Not applicable (***If,Q-4.16´No/Don't know'***)	
4.19	Who checked on your baby's health?<br>(01 month from birth)	01 = Health Personnel / Qualified Doctor<br>02=Nurse/Midwife/Paramedic<br>03 = Community Counselor<br>04 = Community Health and Nutrition Worker<br>05 = Community Health and Nutrition Mobilizer<br><br>06 = Family Welfare Visitor<br>07 = Community Skilled Birth Attendant<br>08 = MA/SACMO<br>09 = Health Assistant<br>10 = Family Welfare Assistant<br>11 = Trained TBA<br>12 = Untrained TBA<br>[illegible]	

5. Breastfeeding Practices and Experiences

Read: Now I would like to ask you some questions about how you have been feeding your <u>NEWBORN</u> from birth until now.

5.1	***How much time after birth did you first put (INFANT NAME) to your breast to feed him/her?***	01 = Immediately (within 5 minutes)<br>02 = Within 6- 15 minutes<br>03 = Within 16- 30 minutes<br>04 = 31-59 minutes<br>05 =1- 2 hours<br>06 = 3-5 hours<br>07 = 6-9 hours<br>08 = 10-12 hours<br>09 = 13-24 hours<br>10 = 25-36 hours	11 = 37-48 hours<br>12 = after 2 days<br>13 = after 3 days<br>14 = 4 days or more<br>77 = Other. Specify<br>__________<br>88 = Don't Know	☐☐
5.2	***How much time after birth was (INFANT NAME) first given colostrum?***	01 = Immediately (within 5minutes)<br>02 = Within 6- 15 minutes<br>03 = Within 16- 30 minutes<br>04 = 31-59 minutes<br>05 =1- 2 hours<br>06 = 3-5 hours<br>07 = 6-9 hours<br>08 = 10-12 hours<br>09 = 13-24 hours<br>10 = 25-36 hours	11 = 37-48 hours<br>12 = after 2 days<br>13 = after 3 days<br>14 = 4 days or more<br>77 = Other. Specify<br>__________<br>88 = Don't Know	☐☐
5.3	***How much time after birth was (INFANT NAME) first given*** any other liquids to drink (such as Sugar Water/Honey/Oil) except breast milk***?***	01 = Immediately (within 5minutes)<br>02 = Within 6- 15 minutes<br>03 = Within 16- 30 minutes<br>04 = 31-59minutes<br>05 =1- 2 hours<br>06 = 3-5 hours<br>07 = 6-9 hours<br>08 = 10-12 hours<br>09 = 13-24 hours<br>10 = 25-36 hours	11 = 37-48 hours<br>12 = after 2 days<br>13 = after 3 days<br>14 = 4 days or more<br>15=Not given<br>77 = Other. Specify<br>__________<br>88 = Don't Know	☐☐
5.4	***At what age was (INFANT NAME) first given breast milk to drink?***	01=Never given breast milk<br>02 = Immediately (within 5minutes)<br>03 = Within 6- 15 minutes<br>04 = Within 16- 30 minutes<br>05 = 31-59 minutes<br>06 =1- 2 hours<br>07 = 3-5 hours<br>08 = 6-9 hours<br>09 = 10-12 hours<br>10 = 13-24 hours<br>11 = 25-36 hours	12 = 37- 48 hours<br>13 = after 2 days<br>14 = after 3 days<br>15 = 4 days or more<br>16=1 week after birth<br>17=2 weeks after birth<br>18=3 weeks after birth<br>19=Not given<br>77 = Other. Specify<br>__________<br>88 = Don't Know	☐☐
5.5	***Has (INFANT NAME) ever been given water to drink?***		01 = Yes<br>02 = No>> Skip to 5.7<br>88 = Don't Know>> Skip to 5.7	☐☐
5.6	***At what age was (INFANT NAME) first given water to drink?***	01 = Never given water<br>02 = Immediately (within5 minutes)<br>03 = Within 6-15 minutes<br>04 = Within16- 30 minutes<br>05 = 31-59minutes<br>06 = 1-2 hours<br>07 = 3-5 hours<br>08 = 6-9 hours	12 = 37hours -1 week after birth<br>13 = 1 weeks after birth<br>14 = 2 weeks after birth<br>15 = 1months after birth<br>16 = 2 months after birth<br>17= 3 months after birth<br>18=Don't give<br>77 = Other. Specify<br>__________	☐☐

5.6	***At what age was (INFANT NAME) first given water to drink?***	09 = 10-12 hours<br>10 = 13-24 hours<br>11 = 25-36 hours	88 = Don't Know<br>99= Not applicable	
5.7	***Has (INFANT NAME) ever been given food to eat?***		01 = Yes<br>02 = No >> Skip to 5.9<br><br>88 = Don't Know>> Skip to 5.9	
5.8	***At what age was (INFANT NAME) first given food to eat?***	01 = Never given food<br>02 = Immediately (within 5minutes)<br>03 = Within6- 15 minutes<br>04 = Within16- 30 minutes<br>05 = 31-59minutes<br>06 =1- 2 hours<br>07 = 3-5 hours<br>08 = 6-9 hours<br>09 = 10-12 hours<br>10 = 13-24 hours<br>11 = 25-36 hours	12 = 37hours-1 week after birth<br>13 = 1 weeks after birth<br>14 =2 weeks after birth<br>15 = 1 months after birth<br>16 = 2 months after birth<br>17=3 months after birth<br>18=Don't give<br>77 = Other. Specify-----------------------------------<br>88 = Don't Know<br>99= Not applicable	

**5.9**	**5.10**	**5.11**	**5.12**	**5.13**	**5.14**
Colostrum is the first milk-like substance, thick, yellow/cream in color, produced during the first few days after birth (0-3 days).<br><br>When (INFANT NAME) was 0-3 days old, what was he/she given to drink?	When (INFANT NAME) was 4 days old, what was he/she given to drink?	When (INFANT NAME) was 2 weeks old, what was he/she given to drink?	When (INFANT NAME) was 1 month old, what was he/she given to drink?	When (INFANT NAME) was 2 months old, what was he/she given to drink?	When (INFANT NAME) was 3 months old, what was he/she given to drink?
Instructions: Please mark all that apply.<br>01 = Breast milk<br>02 = Colostrum<br>03 = Plain Water<br>04 = Animal Milk (Cow, Goat)<br>05 = Sugar Water/Honey/Juice<br>06 = Commercial Infant Formula<br>07 = Yoghurt<br>08 = Luta ("sugi", dilute semolina)<br>88 = Don't Know<br>99= Not applicable<br>77 = Other Liquid. Specify --------------------------					
Other ________	________	________	________	________	________

Read: Now I would like to ask you about any liquids (INFANT NAME) had in the <u>last 24 hours</u>.

5.15	Did (INFANT NAME) drink:		
A	Breast milk?	01 = Yes	________

		02 = No<br>88 = Don't Know	
B	Plain water?	01 = Yes<br>02 = No<br>88 = Don't Know	
C	Sugar Water/Honey/Juice	01 = Yes<br>02 = No<br>88 = Don't Know	
D	Commercially produced infant formula/baby formula?	01 = Yes<br>02 = No<br>88 = Don't Know	
E	Cow's milk?	01 = Yes<br>02 = No<br>88 = Don't Know	
F	Goat's milk?	01 = Yes<br>02 = No<br>88 = Don't Know	
G	Yoghurt?	01 = Yes<br>02 = No<br>88 = Don't Know	
H	Luta ("sugi", dilute semolina)	01 = Yes<br>02 = No<br>88 = Don't Know	
I	Other liquid (aside from breast milk or the liquids just previously named)?	01 = Yes<br>02 = No<br>88 = Don't Know<br><br>If Yes, please specify:<br>__________<br>__________<br>__________<br>__________	

Read: Now I would like to ask you about any foods (INFANT NAME) had in the last 24 hours.

Instructions: Please code '1' if the infant has consumed the food mentioned in the list in past 24 hours, otherwise code '2 Please circle the food name that has been consumed.

5.16	*Food list*	**Example**	*1=Yes, 2=No*
A.	CEREALS	Rice,bread, noodles, biscuits, cookies or any other foods made from rice, wheat , luta	
B.	VITAMIN A RICH VEGETABLES AND TUBERS	pumpkin, carrots or sweet potatoes that are orange inside + other locally available vitamin-A rich vegetables	
C.	WHITE TUBERS AND	White potatoes or foods made from roots.	

	ROOTS		
D.	DARK GREEN LEAFY VEGETABLES	dark green/leafy vegetables locally available vitamin-A rich leaves such as amaranth leaves etc.	
E.	OTHER VEGETABLES	other vegetables e.g. tomato, eggplant	
F.	VITAMIN A RICH FRUITS	ripe mangoes, papaya, jackfruit or other locally available vitamin A-rich fruits	
G.	OTHER FRUITS	other fruits including guava, pineapple, watermelon, melon, orange, apple, grape, banana	
H.	ORGAN MEAT (IRONRICH)	liver, kidney, heart or other organ meats	
I.	FLESH MEATS	beef, lamb, goat, chicken, duck, or other birds	
J.	EGGS	egg	
K.	FISH	fresh or dried fish or shellfish	
L.	LEGUMES, NUTS AND SEEDS	beans, peas, lentils, nuts, seeds or foods made from these	
M.	MILK AND MILK PRODUCTS	Milk (cow's, goat's), cheese, yogurt or other milk products	
N.	OILS AND FATS	oil, fats or butter added to food or used for cooking	
O.	JUNK FOOD	cookies, sweets	

5.17	How many times did (INFANT NAME) eat solid, semisolid or soft foods in the last 24 hours?	00 = 0 times<br>01 = 1 times<br>02 = 2 times<br>03 = 3 times<br>04 = 4 times<br>05 = 5 times<br>06 = 6 times<br>07 = 7 times<br>08 = 8 times or more<br>88 = Don't Know	

Read: Now, I would like to ask you a few questions about bottle feeding (INFANT NAME).

5.18	Have you ever fed (INFANT NAME) using a bottle?	01=Yes<br>02=No » Skip to section 6.1	
5.19	What do you bottle feed (INFANT NAME)?	01=Cow's milk<br>02=Goat's milk<br>03=Commercially produced infant/baby formula<br>04=Breast milk<br>05 = Luta (semolina, or "sugi" diluted with water)<br>06=Water<br>07=Tea<br>08=Fruit juice<br>09=Sugar/water/honey<br>99=Not Applicable(If,Q-5.8 is "no")	

5.20	How often do you bottle feed (INFANT NAME) in a week?	01=7 or more times/week<br>02= 4-6 times/week<br>03=0-3 times/week<br>77=Other. Specify -------------------------------<br>88 = Don't Know<br>99=Not Applicable	
5.21	How many days in the last month did you bottle feed (INFANT NAME)?	-------------------- days in a month<br><br>99=Not Applicable (If,Q-5.8 is "no")	

Study ID

6. Intentions for Introducing Foods

***READ:** Now I would like to ask you some questions about your plans to feed your newborn baby. Please choose the answer that most clearly matches your opinion considering both your plans and the likelihood that you will carry out those plans.*

6.0	***Instructions: In this section, ONLY ask either questions A or B. Based on respondent's answers to 5.9-5.16 decide whether the infant is:***<br>***A) Currently still exclusively breastfed (if Q-5.9-5.14 answer is "01=breast milk" and except 5.15A, all answers including 5.16 are "NO")***<br>***B) Not exclusively breastfed (if 5.9-5.14 is "02-08,88,77 or 5.15B-I and 15.16 any one response is YES)***<br><br>***Please code A or B first.***		01=A<br>02=B

	A – Currently Still Exclusively Breastfed Infants	B - Not Exclusively Breastfed Infants		
6.1	***How long do you plan to give (INFANT NAME) nothing other than breast milk, not even water?***	***How long did you plan to give (INFANT NAME) nothing other than breast milk, not even water?***	00 if <01 month<br>01 month 07 months<br>02 months 08 months<br>03 months 09 months<br>04 months 10 months<br>05 months 11 months<br>06 months 12 months<br><br>>12 months (please specify)<br><br>88=Don't know	
6.2	***When did you decide how long you are going to give (INFANT NAME) nothing other than breast milk, not even water?***	***Skip to 6.3***	01= Before you became pregnant<br>02= During pregnancy<br>03= After the baby's birth<br>04=During the first three months after birth<br>05=Did not make any decision<br><br>88=Don't know<br><br>99= Not Applicable (If Q-6.2 B is not answered)	
6.3	***Skip to 6.5***	***How long do you plan to give (INFANT NAME) breast milk?***	03 month 09 months 15 months<br>04 months 10 months 16 months<br>05 months 11 months 17 months<br>06 months 12 months 18 months<br>07 months 13 months 19 months<br>08 months 14 months 20 months<br><br>>20 months (please specify)<br><br>88=Don't know<br>99= Not Applicable (If Q-6.3 A is not answered)	
6.4	***Skip to 6.5***	***When did you decide how long you are going to give (INFANT NAME) breast milk?***	01= Before you became pregnant<br>02= During pregnancy<br>03= After the baby's birth<br>04=During the first three months after birth<br>05=Did not make any decision<br><br>88=Don't know<br><br>99= Not Applicable (If Q-6.4 A is not answered)	
6.5	***At what age, do you plan to first introduce other liquids (aside from breast milk) to***	***At what age, did you first introduce other liquids (aside from breast milk) to***	00 if < 01 month<br>01 month 07 months<br>02 months 08 months	

	(INFANT NAME)'s diet?	***(INFANT NAME)'s diet?***	03 months 09 months<br>04 months 10 months<br>05 months 11 months<br>06 months 12 months<br><br>>12 months (please specify)<br><br>88=Don't know	
	INSTRUCTIONS: Record the number of ***months*** reported. If '1 month', record '01'. If '>12 months', still record in months. For example, if 1 year and 3 months, enter '15m'<br><br>If <01 month, record number of days AND 00 month.			
6.6	***At what age, do you plan to first introduce other foods (aside from breast milk) to (INFANT NAME)'s diet?***	***At what age, did you first introduce other foods (aside from breast milk) to (INFANT NAME)'s diet?***	00 if < 01 month<br><br>01 month 07 months<br>02 months 08 months<br>03 months 09 months<br>04 months 10 months<br>05 months 11 months<br>06 months 12 months<br><br>>12 months (please specify)<br><br>88=Don't know	
6.7 A	***In the first few days (1-3 days) after you begin to give (INFANT NAME) other foods besides breast milk, how many times a day do you plan to give him/her foods?***	***In the first few days after you began giving (INFANT NAME) other foods besides breast milk, how many times a day did you give (INFANT NAME) foods?***	------------------ Times/Day<br><br>88=Don't know	
6.7 B	***In the first week after you begin to give (INFANT NAME) other foods besides breast milk, how many times a day do you plan to give him/her foods?***	***In the first week after you began giving (INFANT NAME) other foods besides breast milk, how many times a day did you give (INFANT NAME) foods?***	------------------------------ Times/Day<br><br>88=Don't know	
6.7 C	***In the first month after you begin to give (INFANT NAME) other foods besides breast milk, how many times a day do you plan to give him/her foods?***	***In the first month after you began giving (INFANT NAME) other foods besides breast milk, how many times a day did you give (INFANT NAME) foods?***	----------------------------- Times/Day<br><br>88=Don't know	
6.7 D	***In the first three months after you begin to give (INFANT NAME) other foods besides breast milk, how many times a day do you plan to give him/her foods?***	***In the first three months after you began giving (INFANT NAME) other foods besides breast milk, how many times a day did you give (INFANT NAME) foods?***	---------------------------- Times/Day<br><br>88=Don't know	
6.8	***Skip to 6.9***	***In the next 6 months from today, how many times a day do you plan to give (INFANT NAME) foods?***	------------------------------- Times/Day<br><br>88=Don't know<br><br>99 = Not Applicable (If,Q-6.8 "A" is not answered)	
6.9	***When you first begin to give (INFANT NAME) other foods, what types of foods do you plan to give in the first week he/she eats foods?***	***When you first began giving (INFANT NAME) other foods, what types of foods did you first give to (him/her) in the first week he/she eats foods?***<br><br>01 = Yes	Cereals (bread, noodles, biscuits, cookies, or any other foods made from rice, wheat)<br><br>Vitamin A Rich Vegetables and Tubers (pumpkin, carrots, or sweet potatoes that are orange inside + other locally available vitamin	

6.9	***When you first begin to give (INFANT NAME) other foods, what types of foods do you plan to give in the first week he/she eats foods?***	02 = No	A-rich vegetables)<br><br>White Tubers and Roots (White potatoes or foods made from roots)<br><br>Dark Leafy Green Vegetables (dark green/leafy vegetables locally available vitamin-A rich leaves such as amaranth leaves etc)<br><br>Vitamin A Rich Fruits (ripe mangoes, papaya, jackfruit or other locally available vitamin A-rich fruits)<br><br>Other Vegetables (e.g. tomatoes, eggplant)<br><br>Other Fruits (e.g. guava, pineapple, watermelon, melon, orange, apple, grape, banana)<br><br>Organ Meat (iron rich)<br><br>Flesh Meats (beef, lamb, goat, chicken, duck, or other birds)<br><br>Eggs<br><br>Fish (fresh/dried fish or shellfish<br><br>Legumes, Nuts, and Seeds (beans, peas, lentils, nuts, seeds, or foods made from these)<br><br>Milk (cow's, goat's), cheese, yogurt, or other milk products<br><br>Oil, fats, or butter added to food or used for cooking<br><br>Other(s). Specify____________________<br><br>______________________________	
6.10	***Skip to 6.11***	***What additional types of foods do you plan to introduce to (INFANT NAME)'s diet after he/she has been eating for 6 months?***<br><br>01 = Yes<br>02 = No<br><br>99=Not Applicable (If,Q-6.10 "A" is not answered)	Cereals (bread, noodles, biscuits, cookies, or any other foods made from rice, wheat)<br><br>Vitamin A Rich Vegetables and Tubers (pumpkin, carrots, or sweet potatoes that are orange inside + other locally available vitamin A-rich vegetables)<br><br>White Tubers and Roots (White potatoes or foods made from roots)<br><br>Dark Leafy Green Vegetables (dark green/leafy vegetables locally available vitamin-A rich leaves such as amaranth leaves etc)	

			Vitamin A Rich Fruits (ripe mangoes, papaya, jackfruit or other locally available vitamin A-rich fruits)	
			Other Vegetables (e.g. tomatoes, eggplant)	
			Other Fruits (e.g. guava, pineapple, watermelon, melon, orange, apple, grape, banana)	
			Organ Meat (iron rich)	
			Flesh Meats (beef, lamb, goat, chicken, duck, or other birds)	
			Eggs	
			Fish (fresh/dried fish or shellfish	
			Legumes, Nuts, and Seeds (beans, peas, lentils, nuts, seeds, or foods made from these)	
			Milk (cow's, goat's), cheese, yogurt, or other milk products	
			Oil, fats, or butter added to food or used for cooking	
			Do you plan	
			Other(s). Specify____________________	

6.11	***Have you heard of sprinkles or multiple-micronutrient powders (MNP)?***	01 = Yes<br>02 = No>>skip to 7.1<br><br>88 = Don't know >>skip to 7.1	
6.12	***Do you plan to give sprinkles to (INFANT NAME)?***	01 = Yes>>skip to 6.14<br><br>02 = No<br><br>03 = Already give sprinkles>>skip to 6.14<br><br>88 = Don't know>>skip to 7.1<br>99 = Not Applicable	
6.13	***Why not?***<br>***1=Yes,99*** =Not Applicable(If no response)<br><br>***Instructions: Please mark all responses.***	Difficult to find in stores<br><br>Too expensive<br><br>No need to<br><br>Doctor never recommended<br><br>My family does not think necessary<br><br>I don't think it is necessary	

		My friends do not think it is necessary<br><br>Others. Please specify:<br>__________________<br>__________________<br><br>88 = Don't Know<br>99 = Not Applicable (If Q-6.12 = Yes)	
6.14	***Why?***	Good for baby<br><br>Easy to feed sprinkles<br><br>Reasonable price<br><br>Recommended<br><br>Others also use sprinkles<br><br>Others. Please specify:<br>__________________<br>__________________<br><br>88 = Don't Know<br>99 = Not Applicable (If Q-6.11 = No)	

7. Knowledge on Introducing Foods

READ: Now I would like to ask you some general questions regarding feeding your baby foods. After each of the following statements, I will read out options.

Instructions: Read ALL the options.

7.1	***At what age is it recommended that a baby begin to drink other liquids, aside from breast milk?(to be given)***	00= Less than 01 month after baby's birth<br>01 month 07 months<br>02 months 08 months<br>03 months 09 months<br>04 months 10 months<br>05 months 11 months<br>06 months 12 months<br><br>>12 months (please specify)<br><br>88=Don't know<br><br>99= Not Applicable (if no response)	
7.2	***How long after birth is it recommended that a baby begin to eat foods, aside from breast milk? (to be given)***	00= Less than 01 month after baby's birth<br>01 month 07 months<br>02 months 08 months<br>03 months 09 months<br>04 months 10 months<br>05 months 11 months<br>06 months 12 months<br><br>>12 months (please specify)<br><br>88=Don't know<br><br>99= Not Applicable (if no response)	

7.3	***How does a mother know when to introduce solid foods to (INFANT NAME)'s diet?***<br><br>*INSTRUCTIONS: Please record all that apply*<br><br>01 = Yes<br>02 = No<br>99 = Not Applicable (If no response)	When infant is older than 6 months of age<br><br>Infant can sit by him/herself<br><br>Infant can hold up his/her head<br><br>Infant has lost tongue thrust<br><br>Infant has begun teething<br><br>Infant can make chewing motions<br><br>Infant has gained significant weight gain since birth<br><br>Infant can close mouth around spoon<br><br>Infant can control/move tongue around<br><br>Infant shows interest in food<br><br>Infant cries<br><br>Infant is restless<br><br>Mother's breast milk is not enough<br><br>88=Don't know<br><br>77=Other (Specify)____________________	

7.4	***At that time, what are the first foods that are <u>recommended</u> for a baby to eat?***<br><br>*INSTRUCTIONS: Please record all that apply.* First only ask the question and record responses. Then read out other remaining answers and record responses.)<br><br>01 = Yes<br>02 = No<br>99 = Not Applicable (If no response)	Cereals (rice, wheat, jawar, hotchpotch)	
		Legumes (lentils, pulses, beans etc)	
		Foods from animals (egg, meat, fish)	
		Milk and milk products	
		Oil or fat	
		Sugar	
		Vegetables	
		Fruits	
		Nutritional supplements (sprinkles)	
		"Luta" (semolina, or "sugi" in a watery suspension)	
		Other (Specify) ____________	

7.5	***When feeding a meal to a child who is 7-12 months old, what types of foods would you include in a single meal?***<br><br>*INSTRUCTIONS: Please record all that apply.* First only ask the question and record responses. Then read out other remaining answers and record responses.)<br><br>01 = Yes<br>02 = No<br>99 = Not Applicable (***If no response)***	Cereals (rice, wheat, jawar, hotchpotch)	
		Legumes (lentils, pulses, beans etc)	
		Foods from animals (egg, meat, fish)	
		Milk and milk products	
		Oil or fat	
		Sugar	
		Vegetables	
		Fruits	
		Nutritional supplements (sprinkles)	
		"Luta" (semolina, or "sugi" in a watery suspension)	
		Other (Specify) ____________	

READ: Now I would like to ask you a few more questions about feeding your baby foods, in addition to breast milk. For these statements, I will not be reading aloud any options.

7.6	***When an infant begins to eat foods in addition to breast milk, what are the <u>recommended</u> ways that his/her food be prepared?***<br><br>*INSTRUCTIONS: Please record all that apply*	Same food as for family<br>Different food than family<br>Watered down food	

7.6	01 = Yes<br>99 = Not Applicable (If no response by mother)	Pureed<br>Mashed<br>Semi-solid<br>Other (Specify)<br>---<br>---	

7.7	***In general, is it recommended that infants under 6 months of age be given water if the weather is very hot?***	01= Yes<br>02= No<br>88=Don't know	
7.8	***Imagine there is a mother who is having trouble beginning to introduce foods to her infant. What are the recommended ways a mother can try to feed her infant foods, aside from breast milk?***<br>*INSTRUCTIONS: Please record all that apply*<br>01= Yes<br>99=Not applicable (if not responded)	Active encouragement<br>Giving infant his/her own plate<br>Force feeding<br>Introducing new foods one at a time<br>Talk to child while feeding<br>Maintaining eye-to-eye contact<br>Minimize distractions during meals<br>Include a variety of foods slowly<br>Include nutritional supplements<br>Play (as encouragement)<br>Other. Specify ___________________	
7.9	***Do you think others will help you to feed your child?***<br>01= Yes<br>99=Not applicable (if not responded)	Father-in-law<br>Mother-in-law<br>Husband<br>Sister-in-law<br>No, no one will help	

		Others (Specify)	

READ: Now I would like to ask you a few more questions about feeding your baby foods if he/she has diarrhea. After each of the following questions, I will read out options.

7.10	***What is recommended for a mother to do when her child has diarrhea?***<br><br>*INSTRUCTIONS:* ***Please read ALL options, and*** *record all responses.*<br><br>01 = Yes<br>02= No<br>99 = Not Applicable (If no response)	Give syrups<br>Give traditional medicine<br>Treated by doctor<br>Give Zinc<br>ORS<br>Other [Specify]-------------------------------	
7.11	***What is recommended for a mother to do when her child had diarrhea/another illness?***<br><br>*INSTRUCTIONS:* ***Please read ALL options, and*** *record all responses.*<br><br>*PROBE: To clarify between different choices (e.g. less or more than usual?)*<br><br>01 = Yes<br>02 = No<br>99 = Not Applicable (If no response)	Continue breastfeeding>> If No, Skip next two choices (BF less or more than usual)<br>Breastfeed less than usual<br>Breastfeed more than usual<br>Give less foods than usual<br>Give as much foods as usual<br>Give more food than usual<br>Give less liquids than usual<br>Give as much liquids as usual<br>Give more liquids than usual<br>Give treated water<br>Give carrot juice or rice water	
7.12	***What should a mother do (in relation to feeding) AFTER her child has recovered from diarrhea or another illness?***<br><br>*INSTRUCTIONS:* ***Please read ALL options, and*** *record all responses.*<br><br>*PROBE: To clarify between different choices (e.g. less or more than usual?)*<br><br>01 = Yes<br>02 = No<br>99 = Not Applicable (If no response)	Continue breastfeeding>> If No, Skip next two choices (BF less or more than usual)<br>Breastfeed less than usual<br>Breastfeed more than usual<br>Give less foods than usual<br>Give as much foods as usual<br>Give more food than usual	

		Give less liquids than usual<br>Give as much liquids as usual<br>Give more liquids than usual<br>Give nutrient food<br>Give drugs/vitamins<br>Give treated water<br>Give carrot juice or rice water<br>Other [Specify]-------------------------------	

7.13	Who provides you information on breastfeeding?<br>*Instructions: Please record all responses.*<br>01 = Yes<br>99 = Not Applicable (If no response)	Mother<br>Mother in law<br>Husband<br>Elder sister<br>Sister-in-law<br>Health Assistant<br>Family Welfare Assistant<br>MTMSG facilitator<br>Community Counselor<br>Community health and nutrition workers<br>Community health and nutrition mobilizer<br>Neighbor<br>Friend<br>None<br>Other. Specify<br>____________________<br>____________________<br>Don't Know	

Study ID

7.14	Who provides you information on complementary feeding?<br><br>*Instructions: Please record all responses.*<br><br>01 = Yes<br>99 = Not Applicable (If no response)	Mother<br>Mother in law<br>Husband<br>Elder sister<br>Sister-in-law<br>Health Assistant<br>Family Welfare Assistant<br>MTMSG facilitator<br>Community Counselor<br>Community health and nutrition workers<br>Community health and nutrition mobilizer<br>Neighbor<br>Friend<br><br>None<br><br>Other. Specify<br>____________________<br>____________________<br>Don't Know	
7.15	Who provides you information on micronutrient powders (SPRINKLES)?<br><br>*Instructions: Please record all responses.*<br><br>01 = Yes<br>99 = Not Applicable (If no response)	Mother<br>Mother in law<br>Husband<br>Elder sister<br>Sister-in-law<br>Health Assistant<br>Family Welfare Assistant<br>MTMSG facilitator<br>Community Counselor<br>Community health and nutrition workers<br>Community health and nutrition mobilizer<br>Neighbor<br>Friend<br>None<br>Don't Know<br>Other. Specify------------------------------	

7.16	Where do you gain knowledge about breastfeeding?<br><br>*Instructions: Please record all responses.*<br><br>01 = Yes<br>99 = Not Applicable (If no response)	-Counselors<br>- MTMSG<br>- Group education<br>- EPI/GMP<br>- Satellite clinic<br>- Community clinic<br>None<br>- Other. Specify<br>__________<br>__________<br>- Don't Know	
7.17	Where do you gain knowledge about complementary feeding?<br><br>*Instructions: Please record all responses.*<br><br>01 = Yes<br>99 = Not Applicable (If no response)	- Counselors<br>- MTMSG<br>- Group education<br>- EPI/GMP<br>05 = Satellite clinic<br>- Community clinic<br>None<br>Radio<br>TV<br>News Paper<br>Books<br>- Other. Specify<br>__________<br>__________<br>- Don't Know	
7.18	Where do you gain knowledge about micronutrient powders		

7.18	(SPRINKLES)?<br><br>*Instructions: Please record all responses.*<br><br>01 = Yes<br>99 = Not Applicable (If no response)	- Counselors<br>- MTMSG<br>- Group education<br>- EPI/GMP<br>- Satellite clinic<br>- Community clinic<br>None<br>- Other. Specify<br>__________<br>__________<br>- Don't Know	

Study ID

8. Breastfeeding and Complementary Feeding Attitudes

READ: Now I would like to ask you about your intentions regarding feeding infants foods in addition to breast milk. Please consider the following statements about feeding your baby, (INFANT NAME). After I read each statement, please tell me whether you strongly disagree, disagree, agree, strongly agree with, or are neutral about the statement.

		Strongly Agree 1	Agree 2	Neutral 3	Disagree 4	Strongly Disagree 5
8.1	***Eating foods in addition to breast milk is healthy for the baby when he/she is older than 6 months.***	1	2	3	4	5
8.2	***When (INFANT NAME) is one year old, it is best to feed him/her both foods AND provide breast milk.***	1	2	3	4	5
8.3	***Feeding (INFANT NAME) food will make it easier for me to get rest at night.***	1	2	3	4	5
8.4	***Feeding (INFANT NAME) food costs more than just feeding my baby breast milk.***	1	2	3	4	5
8.5	***Feeding (INFANT NAME) nutritional supplements (such as sprinkles) ensures that he/she has the nutrition he/she needs.***	1	2	3	4	5
8.6	***It is easy to find nutritional supplements (such as sprinkles).***	1	2	3	4	5
8.7	***Nutritional supplements (such as sprinkles) are affordable.***	1	2	3	4	5
8.8	***I am able to provide enough breast milk for my baby.***	1	2	3	4	5
8.9	***I am always able to provide enough breast milk for my baby.***	1	2	3	4	5
8.10	***I have someone who is supportive of my breastfeeding.***<br><br>Instructions: If "Disagree" marked >> Skip to 8.12	1	2	3	4	5

8.11	***Who supports your breastfeeding?***<br><br>Instructions: Please mark ALL responses.<br>01 = Yes<br>99 = Not Applicable (If no response)	Husband<br>Mother<br>Mother-in-law<br>Sister<br>Sister-in-law<br>Friend<br>No one supports<br>Other. Specify:	
8.12	***How would you rate your overall breastfeeding experience with (INFANT NAME) on a scale from 1 to 5***. 1 being 'very bad, will never again breast feed', and 5 being 'very good, will continue to breastfeed future children.'	Very Bad 1 — Bad 2 — So-So 3 — Good 4 — Very Good 5	

READ: Now I would like to ask you about who makes decisions about breastfeeding (INFANT NAME).

8.13	***Who makes decisions about up to what age (INFANT NAME) will be (or was) exclusively breastfed?***<br><br>***Instructions: (code '99' if the baby is fed with other food at present)***	***01 = Mother (interviewee)***<br>***02 = Father***<br>***03 = Mother's mother***<br>***04 = Mother-in-law***<br>***05 = Sister***<br>***06 = Sister-in-law***	

		99=Not applicable<br><br>***77 = Other. Specify*** ___________________________	
8.14	***Who makes decisions about up to what age (INFANT NAME) will be (or was) breastfed?***	***01 = Mother (interviewee)***<br>***02 = Father***<br>***03 = Mother's mother***<br>***04 = Mother-in-law***<br>***05 = Sister***<br>***06 = Sister-in-law***<br><br>***99=Not applicable***<br><br>***77 = Other. Specify***_________________	

Study ID | | | |

Read: For each of the following individuals, indicate what they believe is the best way to feed your child after 6 months of age. The options are as follows:<br><br>***The baby should:***<br>***1) Receive only breast milk after 6 months of age.***<br>***2) Receive foods in addition to breast milk after 6 months of age.***<br>***3) Receive only foods (and no breast milk) after 6 months of age.***<br><br>***Please let me know if you would like me to repeat the three options at any point.***<br><br>**If father/mother/mother-in-law died or received no advice from doctor** *please note this by marking "99" as the response*<br>**If respondent does not know what the person thinks** *please note this by marking "88" as the response*

8.15	***My husband thinks I should***	1	2	3	99	88
8.16	***My mother thinks I should***	1	2	3	99	88
8.17	***My mother-in-law thinks I should***	1	2	3	99	88
8.18	***My sister thinks I should***	1	2	3	99	88
8.19	***My doctor thinks I should***	1	2	3	99	88

Read: Now for each of the following individuals, please indicate what they believe is the best way to feed your child at 9 months of age. The options are as follows:<br><br>***The baby should:***<br>***1) Receive only breast milk at 9 months of age.***<br>***2) Receive foods in addition to breast milk at 9 months of age.***<br>***3) Receive foods (and no breast milk) at 9 months of age.***<br><br>***Please let me know if you would like me to repeat the three options at any point.***<br><br>**If father/mother/mother-in-law died or received no advice from doctor** *please note this by marking "99" as the response*<br>**If respondent does not know what the person thinks** *please note this by marking "88" as the response*

8.20	***My husband thinks I should***	1	2	3	99	88
8.21	***My mother thinks I should***	1	2	3	99	88
8.22	***My mother-in-law thinks I should***	1	2	3	99	88
8.23	***My sister thinks I should***	1	2	3	99	88
8.24	***My doctor thinks I should***	1	2	3	99	88

Read: Now for each of the following individuals, please indicate what they believe is the best way to feed your child at 9 months of age. The options are as follows:<br><br>***At 9 months of age, the baby should:***<br>***1) Receive only breast milk at 9 months of age.***<br>***2) Receive foods without sprinkles(multiple micronutrient powder (MNP)) in addition to breast milk***<br>***3) Receive food with sprinkles in addition to breast milk***<br>***4) Receive only foods without sprinkles***<br>***5) Receive only food with sprinkles***<br><br>***Please let me know if you would like me to repeat the five options at any point.***<br><br>**If father/mother/mother-in-law died or received no advice from doctor** *please note this by marking "99" as the response*<br>**If respondent does not know what the person thinks** *please note this by marking "88" as the response*

8.25	***My husband thinks I should***	1	2	3	4	5	99	88
8.26	***My mother thinks I should***	1	2	3	4	5	99	88
8.27	***My mother-in-law thinks I should***	1	2	3	4	5	99	88

8.28	***My sister thinks I should***	1	2	3	4	5	99	88
8.29	***My doctor thinks I should***	1	2	3	4	5	99	88

Read: Now I would like to ask for you to please tell me the degree to which you agree or disagree with the following statements.

1 = Strongly Agree 2 =Agree 3 = Neutral 4 = Disagree 5 = Strongly Disagree

8.30	***I am able to give my baby foods in addition to breast milk.***	1	2	3	4	5
8.31	***I know how to give my baby foods in addition to breast milk.***	1	2	3	4	5
8.32	***I am determined to give my baby foods in addition to breast milk.***	1	2	3	4	5
8.33	***I won't need help to give my baby foods in addition to breast milk.***	1	2	3	4	5
8.34	***Giving my baby foods in addition to breast milk is easy.***	1	2	3	4	5
8.35	***I am confident I can give my baby foods in addition to breast milk.***	1	2	3	4	5
8.36	***I am confident I can give my baby foods with micronutrient powders in addition to breast milk***	1	2	3	4	5

READ: Now I would like to ask you about you and people in your family or community.
How much do you agree with the following statements?1 Please tell me whether you you agree, disagree, or are unsure.
Since you delivered your new baby…

8.37	***… I have someone to help me if I am sick or need to rest***	01 = Agree<br>02 = Disagree<br>03 = Unsure	99=Not applicable<br>77 = Other.<br>Specify________________	
8.38	***… I have someone to take me to the clinic or doctor's office***	01 = Agree<br>02 = Disagree<br>03 = Unsure	99=Not applicable<br>77 = Other.<br>Specify________________	
8.39	***… I have someone to talk with about my problems***	01 = Agree<br>02 = Disagree<br>03 = Unsure	99=Not applicable<br>77 = Other.<br>Specify________________	
8.40	***… I have someone to help me if I am tired and feeling frustrated with my new baby***	01 = Agree<br>02 = Disagree<br>03 = Unsure	99=Not applicable<br>77 = Other.<br>Specify________________	

READ: Now I would like to ask you 2 additional questions, which are still about you and people in your family or community. Again, I will first read a statement. Please tell me whether you you agree, disagree, strongly agree, strongly disagree, or are unsure. 2

8.41	***In general, I can trust the majority of people in my community.***	01 = Strongly Agree<br>02 = Agree<br>03 = Unsure<br>04 = Disagree<br>05 = Strongly Disagree	99=Not applicable<br>77 = Other.<br>Specify________________	
8.42	***I feel as though I am a part of this community.***	01 = Strongly Agree<br>02 = Agree<br>03 = Unsure<br>04 = Disagree<br>05 = Strongly Disagree	99=Not applicable<br>77 = Other.<br>Specify________________	

[1]Adapted from Pregnancy Risk Assessment Monitoring System and
Lippman et al. (2009) Social-Environmental Factors and Protective Sexual Behavior among Sex Workers: The *Encontros* Intervention in Brazil. Amer J Public Health, 99(11), 1-11.
[2] Adapted from Lippman et al. (2009) Social-Environmental Factors and Protective Sexual Behavior among Sex Workers: The *Encontros* Intervention in Brazil. Amer J Public Health, 99(11), 1-11 and SASCAT tool: De Silva and Harpham (2007). Maternal social capital and child nutritional status in developing countries. Health & Place, 13, 341-355.

Study ID

9. Maternal Depression Screener[3]

Read: Now I would like to ask you a few questions about you have been feeling since giving birth to (INFANT NAME). Specifically, I would like for you to now recall how you have been feeling IN THE PAST 2 WEEKS, not just how you feel today.

Instructions: Please read aloud the 4 answer choices before the respondent answers.

READ: Over the last 2 weeks, how often have you been bothered by any of the following problems:

9.1 ***Feeling down, depressed, or hopeless***	01 = Not at all<br>02 = Several days<br>03 = More than half of the days<br>04 = Nearly every day<br><br>05 = Other. Specify ___________________<br>88 = Don't Know	
9.2 ***Little interest or pleasure in doing things***	01 = Not at all<br>02 = Several days<br>03 = More than half of the days<br>04 = Nearly every day<br><br>05 = Other. Specify ___________________<br>88 = Don't Know	

[3] Gjerdingen D, Crow S, McGovern P, Miner M, Center B. Postpartum depression screening at well-child visits: validity of a 2-question screen and the PHQ-9.Ann Fam Med. 2009;7(1):63-70.
Kroenke K, Spitzer RL, Williams JB. The Patient Health Questionnaire-2: validity of a two-item depression screener. Med Care. 2003;41(11):1284-92.

10. Infant Health

READ: Now I would like to ask about (INFANT NAME)'s health in the past two weeks.

10.1	Did (INFANT NAME) receive a vitamin A dose (like this/any of these) since birth?<br><br>Show common types of capsules.	01 = Yes<br>02 = No<br>88 = Don't Know	
10.2	Has (INFANT NAME) had diarrhea in the last 2 weeks?	01 = Yes<br>02 = No >> Skip to 9.13<br>88 = Don't Know>> Skip to 9.13	
10.3	While (INFANT NAME) had diarrhea, was he/she given breast milk?	01 = Yes<br>02 = No >> Skip to 9.5<br>88 = Don't Know>> Skip to 9.5	
10.4	While (INFANT NAME) had diarrhea, how much breast milk was he/she given to drink?<br><br>Was he/she given less than usual breast milk to drink, about the same amount, or more than usual to drink?<br><br>IF LESS, PROBE: Was he/she given much less than usual to drink or somewhat less?	01 = Much Less<br>02 = Somewhat Less<br>03 = About the Same<br>04 = More<br>88 = Don't Know<br>99=Not applicable	
10.5	While (INFANT NAME) had diarrhea, was he/she given liquids (aside from breast milk) to drink?	01 = Yes<br>02 = No >> Skip to 9.7<br>88 = Don't Know>> Skip to 9.7	
10.6	While (INFANT NAME) had diarrhea, how much other liquids (aside from breast milk) was he/she given to drink?<br><br>Was he/she given less than usual other liquids to drink, about the same amount, or more than usual to drink?<br><br>IF LESS, PROBE: Was he/she given much less than usual to drink or somewhat less?	01 = Much Less<br>02 = Somewhat Less<br>03 = About the Same<br>04 = More<br>05= No drinks given<br>88 = Don't Know<br>99=Not applicable	
10.7	When (INFANT NAME) had diarrhea, was he/she given less than usual to eat, about the same amount, more than usual to eat, or nothing to eat?<br><br>Instructions: ONLY ask if the infant is not exclusively breastfed. If the infant is still exclusively breastfed, mark '99'/'Not Applicable'<br><br>IF LESS, PROBE: Was he/she given much less than usual to eat or somewhat less?	01 = Much Less<br>02 = Somewhat Less<br>03 = About the Same<br>04 = More<br>05 = Nothing to Eat<br>88 = Don't Know<br>99 = Not Applicable	

10.8	When (INFANT NAME) had diarrhea, was he/she given multiple micronutrient powder (SPRINKLES) with food?	01 = Yes<br>02 = No >> Skip to 9.13<br>88 = Don't Know >> Skip to 9.13<br>99 = Not Applicable	
10.9	How many sachets of multiple micronutrient powder (SPRINKLES) was (INFANT NAME) given while he/she had diarrhea?	--------------------- no. of sachets<br><br>88 = Don't Know<br>99 = Not Applicable	
10.10	Did you seek advice or treatment when (INFANT NAME) has diarrhea?	01 = Yes<br>02 = No >> Skip to 9.13<br>88 = Don't Know >> Skip to 9.13<br>99 = Not Applicable	
10.11	What was the advice or treatment received?<br><br>Instructions: Please record all responses.	01 = ORS<br>02 = zinc<br>03 = ORS + zinc<br>04 = Drinking more liquids (aside from breast milk)<br>05 = Drinking less liquids (aside from breast milk)<br>06 = Drinking more breast milk than usual<br>07 = Drinking less breast milk than usual<br>08 = Antibiotics<br>88 = Don't Know<br>99=Not applicable	
10.12	Where did you seek advice or treatment from?	01 = Health Personnel / Qualified Doctor<br>02 = Nurse/Midwife/Paramedic<br><br>03 = Community Counselor<br>04 = Community Health and Nutrition Worker<br>05 = Community Health and Nutrition Mobilizer<br><br>06 = Family Welfare Visitor<br>07 = Community Skilled Birth Attendant<br>08 = MA/SACMO<br>09 = Health Assistant<br>10 = Family Welfare Assistant<br>11 = Trained TBA<br>12 = Untrained TBA<br>13 = Unqualified Doctor<br>77 = Other(Specify) ________________<br><br>88 = Don't know<br>99=Not applicable	
10.13	Has (INFANT NAME) been ill with a fever at any time in the last 2 weeks?	01 = Yes<br>02 = No<br><br>88 = Don't Know	
10.14	Has (INFANT NAME) had an illness with a cough at any time in the last 2 weeks?	01 = Yes<br>02 = No >> Skip to 9.17<br>88 = Don't Know>> Skip to 9.17	

10.15	When (INFANT NAME) had an illness with a cough, did he/she breathe faster than usual with short, rapid breaths or have difficulty breathing?	01 = Yes<br>02 = No >> Skip to 9.17<br>88 = Don't Know>> Skip to 9.17<br>99=Not applicable	
10.16	Was the fast or difficult breathing due to a problem in the chest or to a blocked or runny nose?	01 = Chest only<br>02 = Nose only<br>03 = Both<br>77 = Other _________Specify<br>88 = Don't Know<br>99=Not applicable	
10.17	Is (INFANT NAME) still sick with a fever/cough?	01 = Fever only<br>02 = Cough only<br>03 = Both fever and cough<br>04 = No, neither***>> skip to Q 10.1***<br>88 = Don't Know<br>99=Not applicable ***(If,Q-9.13, 9.14 answer "no")***	
10.18	Did you give breast milk when (INFANT NAME) had fever/cough?	01 = Yes<br>02 = No >> Skip to 10.1<br>88 = Don't Know>> Skip to 10.1	
10.19	When (INFANT NAME) had fever/cough, how much breast milk was given to the child?<br><br>Was he/she given less than usual to breast milk, about the same amount, more than usual to breast milk?<br><br>IF LESS, PROBE: Was he/she given much less than usual to eat or somewhat less?	01 = Much Less<br>02 = Somewhat Less<br>03 = About the Same<br>04 = More<br>05 = Nothing to drink<br>88 = Don't Know<br>99 = Not Applicable	
10.20	When (INFANT NAME) had fever/cough, was he/she given less than usual to eat, about the same amount, more than usual to eat?<br><br>Instructions: ONLY ask if the infant is not exclusively breastfed. If the infant is still exclusively breastfed, mark '99'/'Not Applicable'<br><br>IF LESS, PROBE: Was he/she given much less than usual to eat or somewhat less?	01 = Much Less<br>02 = Somewhat Less<br>03 = About the Same<br>04 = More<br>05 = Nothing to drink<br>88 = Don't Know<br>99 = Not Applicable	
10.21	Did you seek advice or treatment when (INFANT NAME) has diarrhea?	01 = Yes<br>02 = No >> Skip to 10.1<br>99=Not applicable	
10.22	What was the advice or treatment received?<br><br>Instructions: Please record all responses.	01 = Acetaminophen<br>02 = Ibuprofen<br>03 = Oral antibiotics<br>04 = Shot<br>05 = Both oral antibiotics and shot<br>06 = Intravenous (IV) fluids<br>07 = Cough medicine	

		08 = Aspirin<br><br>77 = Other. Specify:____________________<br>88 = Don't Know<br><br>99=Not applicable	
10.23	Where did you seek advice or treatment from when child had fever/cough?	01 = Health Personnel / Qualified Doctor<br><br>02 = Nurse/Midwife/Paramedic<br><br>03 = Community Counselor<br><br>04 = Community Health and Nutrition Worker<br><br>05 = Community Health and Nutrition Mobilizer<br><br>06 = Family Welfare Visitor<br><br>07 = Community Skilled Birth Attendant<br><br>08 = MA/SACMO<br><br>09 = Health Assistant<br><br>10 = Family Welfare Assistant<br><br>11 = Trained TBA<br><br>12 = Untrained TBA<br><br>13 = Unqualified Doctor<br><br>77 = Other(Specify) ________________<br><br>88 = Don't know<br><br>99=Not applicable	

Study ID

11. Anthropometrics

Read: Now I would like to take your height and weight measurements. Also, a finger prick blood sample will be taken in order to check the amount of iron that is in your blood. Please know that this is a common, standard test for iron, which requires only a small drop of blood.

MEASUREMENTS FOR MOTHER		
11.1	HEIGHT IN CENTIMETERS	CM ___ ___ ___ . ___
11.2	WEIGHT IN KILOGRAMS	KG ___ ___ . ___
11.3	HEMOGLOBIN IN FINGERPRICK BLOOD SAMPLE	gm/dL ___ ___ ___ .
11.4	STATUS OF HEIGHT, WEIGHT, AND HEMOGLOBIN MEASUREMENTS<br>Measured ……... 1<br>Not Present…… 2<br>Refused all…… 3<br>Other ………… 7	___ ___

Read: Now I would now like to take length, weight, head circumference, and iron measurements of (INFANT NAME) A finger prick blood sample will also be taken in order to check the amount of iron that is in your blood. Again, this is a common, standard procedure that requires only a small drop of blood.

ANTHROPOMETRIC MEASURES AND IRON STATUS FOR CHILD (3 MONTHS)			
11.5	RECORD NAME OF INFANT FROM 4.1		NAME ____________
11.6	RECORD SEX OF INFANT FROM 4.2		01=Male<br>02=Female ___ ___
11.7	WEIGHT IN KILOGRAMS	Caretaker with child	KG ___ ___ . ___
		Only mother	KG ___ ___ . ___
11.8	LENGTH IN CENTIMETERS<br>Instructions: Measurement should be taken when infant is lying down as flat as possible on his/her back.		CM ___ ___ ___ . ___
11.9	HEAD CIRCUMFERENCE IN CENTIMETERS		CM ___ ___ ___ . ___
11.10	HEMOGLOBIN IN FINGERPRICK BLOOD SAMPLE		gm/dL ___ ___ ___ . ___

Study ID | | | |

11.11	STATUS OF HEIGHT, WEIGHT, AND HEMOGLOBIN MEASUREMENTS<br><br>Measured ……... 1<br>Not Present…… 2<br>Refused all…… 3<br>Other ………… 7	

Study ID

Was anyone other than the respondent present at the time of interview?
01=Yes
02=No

If 'Yes' mention

Husband

Father-in-law//Mother-in-law

Daughter/Son

Elder sister-in-law/Sister–in-law

Uncle/Aunt

Other household members

Neighbor (Name, relation)

Notes:

Name of Interviewer ______________________ Code

Name of Quality Controller ______________________ Code

Date:---------------------------
