# Supplementary material for: Maternal knowledge and attitudes towards complementary feeding in relation to timing of its initiation in rural Bangladesh
Source: BMC Nutr. 2019 Jan 30;5:7. doi: 10.1186/s40795-019-0272-0 (PMC7050709; doi:10.1186/s40795-019-0272-0)
Supplement: Supplementary file 2 — Questionanaire used at child age 9 mo. (PDF 1192 kb) [file 40795_2019_272_MOESM2_ESM.pdf]

Mother Study ID | | | | |

Monitoring growth and nutritional status of children in rural Bangladesh
Nine Month Household Questionnaire

1. Identification

Read: To begin with, I would like to collect some basic identification information from you.

	Date of Interview:	Date __ __ Month __ __ Year __ __ __ __	
	Mobile no:<br>*Instructions: Please fill these numbers in from the 3 Month Questionnaire. Confirm that these numbers have not changed, however update if needed.*	Own __ __ __ __ __ __ __ __ __ __ __<br>Request __ __ __ __ __ __ __ __ __ __ __<br>Name:	
	Instructions: Please confirm the mother's name and study ID from the 3 Month Questionnaire (Q-1.1).		
1.1	**MOTHER** (Interviewee) **Name:**______________________	Mother Study ID __ __ __ __	
1.2	***Instructions****: Before interview, please check 3 Month Questionnaire and record name (Q-4.1). During interview, please confirm this is correct.*<br>*IF infant is a twin, triplet, or multiple birth, please also complete a* **MULTIPLE BIRTH SUPPLEMENT** ***after*** *completing this questionnaire for the infant who was first-born.*	**INFANT Name:**<br>______________________	
1.3	***Instructions****: Before interview, please check 3 Month Questionnaire and record gender (Q-4.2). Please circle gender.*	Sex of infant:<br>M F	

1.4	***Is (INFANT NAME) alive today?***	01 = Yes<br>02 = No **>> Skip to VERBAL AUTOPSY SUPPLEMENT**<br>**(Do NOT complete rest of this questionnaire)**<br>88 = Don't know<br>99 = Declined to answer	__ __

1.5	Are you planning to move or be living at another place, different than the place you are living at now?	01 = Yes<br>02 = No >> Skip to 1.7<br>88 = Don't know	__ __
1.6	How can we best reach you in 6 months?<br>Instructions: Write down new address, or probe for phone number that will not change in 6 months.	________________________<br>________________________<br>__ __ __ __ __ __ __ __ __ __ __	
1.7	Are you currently working for income?	01 = Yes<br>02 = No >> Skip to 2.1<br>99 = Not Applicable	__ __
1.8	What type of work are you doing?	*Instructions: Code for different types of occupations below.*	__ __

Code for 1.8 - Types of Occupations
01=Professional/technical (Doctor, engineer, lawyer, teacher, economist, agriculturist), 02=Large business (≥10,000/ Taka invested), 03=Small business (<10,000/ Taka invested), street vendors, 04=Blue collar services:(Factory worker, industry worker, garment worker), 05=White collar services: (Officer, manager, administrator, clerk), 06=Skilled worker (Driver, potter, black smith, gold smith, carpenter, mason, plumber, mechanic), 07=Un-skilled worker (Boatman, fisherman), 08=Day laborer (Rickshaw/cart puller, construction worker, daily wage labor), 09=Farmer/share cropper, 10=Domestic maid/house maid, 11=House wife, 12=Overseas employment, 13=Beggar, 14=Unemployed, 15=Student, 16=Old aged/inactive, 17=Household work, 99=Not applicable, 77=Other (specify)____________________

2. Changes in Demographics and Socio-economic Status[1]

Read: Several months ago, we asked about the people usually living in your household. Now I would like to ask you again about whether there have been any changes in the number of people living in your household, including any deaths.

2.1	***Does (FAMILY MEMBER NAME) still usually live in your household? Also be sure to tell me if you are the person, or if the person has died.***<br><br>01 = Yes<br>02 = No<br>03 = No, person has died<br>04 = Yes, that person is the woman herself.<br>88 = Don't Know<br><br>***Instructions: BEFORE THE SURVEY, please fill out all members of the household listed in the 3-month questionnaire (Section 2.1), including the infant(s). During the survey, read out each member listed in the baseline questionnaire and record who is still there or who is no longer part of the household.***<br><br>***When the name read out is the woman herself, please enter '04' as the code.***<br><br>***Read: Please be sure to tell me if the name I read is your name.***	Line No.	Family Member Name	
		01		
		02		
		03		
		04		
		05		
		06		
		07		
		08		
		09		
		10		
		11		
		12		
		13		
		14		

[1] Adapted from Bangladesh Demographic and Health Survey (BDHS) 2007.

Mother Study ID | | | | |

2.2 Household Schedule

Read: We would like to now include any additional people in your household who were not previously recorded. Please give me the names of <u>ONLY ADDITIONAL</u> people who now usually live in your household.

Line no.	Usual resident and visitors	Relationship to head of household	Sex	Residence		Age	Marital Status	Ever attended school	Level of school attended	Current school attendance	Current paid work status	Current type of work
1	2	3	4	5		6	7	8	9	10	11	12
	-------------------------------<br>List the names and record the relationship and sex for each person.	What is the relationship of (NAME) to the head of the household?<br>----------<br>*See codes below. Include Relationship Code*	Is (NAME) male or female?<br>-------<br>1=male,<br>2=female	Does (NAME) usually live here?<br>-------<br>1=Yes<br>2=No	Did (NAME) stay here last night?<br>-------<br>1=Yes<br>2=No	How old is (NAME)?<br>-------<br>(complete year) *If age is less than 1 year write '00'*<br>88=Don't Know	What is (NAME) current marital status?<br>------------<br>01=currently married<br>02=divorced /separated/ deserted/ widowed<br>03=never-married<br>88=Don't Know,	Has (NAME) ever attended school?<br>---------<br>01=Yes<br>02=No<br>88=Don't Know, `	What is the level of school (NAME) has last attended?<br>-----------<br>*Insert exact number of years completed*<br>33=Can't signature,<br>44=Can signature only,<br>55=Religious education only<br>66=Never went to school<br>88=Don't know<br>99=Not Applicable	Is (NAME) currently attending school?<br>--------<br>01=Yes<br>02=No<br>88=Don't Know,<br>99=Not Applicable	Is (NAME) currently working?<br>--------------<br>01=Yes<br>02=No<br>88=Don't Know,<br>99=Not Applicable no need	What type of work is (NAME) currently doing?<br>-------<br>Use codes given below<br>99= Not Applicable (in case of a child)
01		☐☐	☐	☐	☐	☐☐	☐☐	☐☐	☐☐	☐☐	☐☐	☐☐
02		☐☐	☐	☐	☐	☐☐	☐☐	☐☐	☐☐	☐☐	☐☐	☐☐

03												

Codes for question no. 3: Relationship with the household head

01 = Newborn Infant #1
02 = Newborn Infant #2
03 = Newborn Infant #3
04 = Household head
05 = Spouse
06 = Daughter/son
07 = Son-in-law/daughter-in-law
08 = Grand son/grand daughter
09 = Father/mother
10 = Father-in-law/mother-in-law
11 = Brother-in-law/sister-in-law
12 = Other relative
13= Foster child/step child
14=Non-relative
15=Brother/sister
88=Don't know
99 = Other. Specify ______________________________

Codes of occupation for question no. 12

01=Professional/technical (Doctor, engineer, lawyer, teacher, economist, agriculturist), 02=Large business (≥10,000/ Taka invested), 03=Small business (<10,000/ Taka invested), street vendors, 04=Blue collar services: (Factory worker, industry worker, garment worker), 05=White collar services: (Officer, manager, administrator, clerk), 06=Skilled worker (Driver, potter, black smith, gold smith, carpenter, mason, plumber, mechanic), 07=Un-skilled worker (Boatman, fisherman), 08=Day labor (Rickshaw/cart puller, construction worker, daily wage labor), 09=Farmer/share cropper, 10=Domestic maid/house maid, 11=House wife, 12=Overseas employment, 13=Beggar, 14=Unemployed, 15= Student, 17= Aged, 18= Household task, 99=Not Applicable, 77=Other (specify)____________________

Read: Now I would like to just make sure that we have included everyone in our list of people in your household.

2.3	***Have we now included all other people in your household in our listing?***<br><br>***Instructions: If "No", complete detailed Household Schedule Information below.***	01 = Yes<br>02 = No<br><br>88= Don't Know	

2.4	***(MOTHER NAME), what is your current marital status?***<br><br>***Instructions: If married, please ask which wife she is.***	01 = Married, 1st wife<br>02 = Married, 2nd wife<br>03 = Married, 3rd wife<br>04 = Married, 4th wife<br><br>05 = Single, never married<br>06 = Single, separated<br>07 = Single, widowed<br>08 = Single, divorced<br>09 = Deserted	10 = Other. Please Specify<br>____________<br>____________<br>____________<br>88 = Declined to answer<br>99 =Not Applicable	

3. Food Security / Maternal Diet and Nutrition[2]

Read: Now I would like to ask you some questions about your family's habits relating to food.

Q-01.	During the last 30 d, at what interval has your household purchased rice?	1. Did not buy<br>2. 1–3 times the last 30 d<br>3. Once in 7 d<br>4. 2–3 times in 7 d<br>5. At least 4–5 times in 7 d	☐
Q-02.	During the last 30 d, at what interval has your household purchased "kanchabajar"? (Note: "kanchabajar" refers to shopping of perishable food items such as vegetables, fish and meat.)	1. Did not buy<br>2. 1–3 times the last 30 d<br>3. Once in 7 d<br>4. 2–3 times in 7 d<br>5. At least 4–5 times in 7 d	☐
Q-03.	During the last 30 d, how many times a day did cooking usually take place in your household?	1. Never<br>2. Once a day<br>3. Twice a day<br>4. Three times a day<br>5. Four times or more	☐
Q-04.	During the last 30 d, has your household helped others with cash or food items (like rice) for enabling them to make a meal? (If the girl/woman is poor, tell her that we need to ask this question of everybody, so she does not mind.)	1 = Yes<br>2=No	☐
Q-05.	During the last 30 d, how often has your household had to borrow from others to make a meal? (If the woman is rich, tell her that we need to ask this question of everybody, so she does not mind.)	1. Never >> Skip to Q-07<br>2. 1–3 times in the last 30 d<br>3. Once in 7 d<br>4. 2–3 times in 7 d<br>5. At least 4–5 times in 7 d	☐
Q-06.	Have you paid back or do you think you can pay back?	1 = Yes<br>2 = No	☐
Q-07.	Has your household lent money to others?	1. Yes<br>2. No >> Skip to Q-09	☐
Q-08.	Have you been paid back or do you think you will be paid back?	1 = Yes<br>2 = No	☐
Q-09.	During the last 30 d, how many times on average have you had a fulfilling meal in a day?	1. One time<br>2. Two times<br>3. Three times<br>4. Four times	☐
Q-10.	During the last 30 d, how often has it happened that you could NOT eat as many fulfilling meals as you would like to have done?	1. Never<br>2. Less than once in 7 d<br>3. Once in 7 d<br>4. 2–3 times in 7 d<br>5. At least 4–5 times in 7 d	☐
Q-11.	For the last 30 d, did you usually have snacks in between meals?	1.No<br>2. Once or twice<br>3. Three times or more	☐
Q-12.	For the last 30 d, how often did you have fish?	1.Not once<br>2.Less than once in 7 d<br>3.Once in 7 d<br>4.2–3 times in 7 d<br>5.At least 4–5 times in 7 d	☐
Q-13.	During the last 30 d, how often have you had to eat rice with just chili and salt?	1.Did not have to<br>2.Less than once in 7 d<br>3.Once in 7 d<br>4.2–3 times in 7 d<br>5.At least 4–5 times in 7 d	☐

[2] ICDDR,B (also in 3-Month Questionnaire)

Mother Study ID | | | | |

<u>Dietary Diversity</u>
Individual Dietary Diversity Questionnaire (IDDS)[3]

Instructions:

Q-3.1:	***Please record '01' if the mother has consumed the food mentioned in the list in past 24 hours, otherwise record '02.' Circle the food name that has been consumed. COMPLETE ALL OF Q-3.2 BEFORE BEGINNING Q-3.3.***
Q-3.2:	***Only for each type of the food that was eaten, ask about how often the food was eaten.***

Read: Now I would like to ask you some questions regarding your diet and nutrition. I would like to record any liquids or foods that you had in the past 24 hours.

	Food list	*Examples*	*Q-3.1* *In the last 24 hours, did you eat (FOOD)?* *01=Yes* *02=No*	*Q-3.2* *How many times in the last 24 hours did you eat (FOOD)?* *# of times/day* *8 = Don't Know* *9 = Not Applicable (Not eaten)*
A.	CEREALS - RICE	Rice, smashed rice/rice gruel, bread, noodles, biscuits, or any other foods made from wheat or rice		
B.	CEREALS - OTHER	Maize, maize porridge, sorghum, millet, pasta, bread and other cereals		
C.	VITAMIN A RICH VEGETABLES AND TUBERS	pumpkin, carrots or sweet potatoes that are orange inside + other locally available vitamin-A rich vegetables		
D.	WHITE TUBERS AND ROOTS	White potatoes or foods made from roots.		
E.	DARK GREEN LEAFY VEGETABLES	dark green/leafy vegetables locally available vitamin-A rich leaves (e.g. amaranth leaves)		
F.	OTHER VEGETABLES	other vegetables e.g. tomato, eggplant		
G.	VITAMIN A RICH FRUITS	ripe mangoes, papaya, jackfruit or other locally available vitamin A-rich fruits		
H.	VITAMIN C RICH FRUITS	Orange, papaya or other locally available vitamin C-rich fruits		

[3] Adapted from existing ICDDR,B IDDS (also in 3-Month Questionnaire)

I.	OTHER FRUITS	Other fruits (e.g. guava, pineapple, watermelon, melon, orange, apple, grape, banana)		
J.	ORGAN MEAT (IRON RICH)	liver, kidney, heart or other organ meats		
K.	FLESH MEATS	beef, lamb, goat, chicken, duck, or other birds		
L.	EGGS	Egg		
M.	FISH	Fresh or dried fish or shellfish		
N.	LEGUMES, NUTS AND SEEDS	beans, peas, lentils, nuts (e.g. groundnuts, cashews) , seeds or foods made from these		
O.	MILK AND MILK PRODUCTS	milk (cow's, goat's), cheese, yogurt or other milk products		
P.	OILS AND FATS	oil, fats or butter added to food or used for cooking		
Q.	JUNK FOOD	cookies, cakes, biscuits, chips, sweets, samocha		
R.	CONDIMENTS			
S.	NUTRITIONAL SUPPLEMENTS	*Pushtika* (Sprinkles/MNP)		

3.3	***Are you currently taking any iron tablets or iron syrup?***	01 = Yes<br>02 = No<br>88 = Don't know	
3.4	***In the past 6 months, did you take any iron tablets or iron syrup?***	01 = Yes<br>02 = No >> Skip to 4.1<br>88 = Don't know	
3.5	***In the past 6 months, how many months or days did you take iron tablets?***	# of months OR<br># of days<br>Months<br>09 = Other. Please specify:<br>____________________<br>Days<br>88 = Don't know	

4. Family Planning

Read: Now I would like to ask you some questions regarding family planning.

4.1	Has your menstrual period returned since the birth of (INFANT NAME)?	01 = Yes<br>02 = No >> Skip to 4.3<br><br>88 = Don't Know	
4.2	How long ago did your last menstrual period start?<br><br>***Instructions:*** If mother cannot remember, use local calendar to approximate.	Days Ago<br>Weeks Ago<br>Months Ago<br>Years Ago<br><br>88 = Don't Know<br>99 = Before last birth	
4.3	Are you pregnant now?	01 = Yes<br>02 = No<br><br>88 = Don't know/Unsure	
4.4	If it is up to you, are you planning to become pregnant or have another baby?	01 = Yes<br>02 = No >> Skip to 4.7<br><br>88 = Don't know	
4.5	If it could be up to you, when would you like to become pregnant again?	00 = As soon as possible<br>01 = 1 – 6 months<br>02 = 7 – 12 months<br>03 = 13—18 months<br>04 = 19 – 24 months<br>05 = >2 to 3 years<br>06 = >3 to 4 years<br>07 = >4 to 5 years<br>08 = > 5 years<br>09 = 9 Months<br><br>***Instructions****: If other, please record # of months.*<br><br>88 = Don't know	
4.6	How likely do you think you will actually become pregnant when you would like to, approximately about ___ months from today? Very likely, likely, not sure, unlikely, or very unlikely?	01 = Very likely<br>02 = Likely<br>03 = Not sure<br>04 = Unlikely<br>05 = Very unlikely<br><br>88 = Don't know	
4.7	Do you currently use any type of contraceptive method?	01 = Yes<br>02 = No	

4.7	Do you currently use any type of contraceptive [illegible]	01 = Yes<br>[illegible]	
4.8	What methods of contraception are you currently using?<br><br>01= Yes<br>99=Not Applicable (If not response)<br><br>***Instructions:*** Please record ALL responses.	<u>Any traditional methods</u><br>Azal/Safe period<br><br>Kabiraji/Herbal treatments<br><br>"LAM" (Lactation Amenorrhea Method)<br><br><u>Any temporary modern methods</u><br>Pill<br><br>Condom<br><br>Injection<br><br>IUD<br><br>Norplant<br><br>Female sterilization<br><br>Male sterilization<br><br>Not currently using any method<br><br>Don't Know<br><br>Other. Specify: ______________	
4.9	Do you think you will use a contraceptive method to delay or avoid pregnancy at any time in the future?	01 = Yes<br>02 = No<br><br>88 = Don't Know	
4.10	Have you ever talked to your husband about family planning in the last three months?	01 = Yes<br>02 = No >> Skip to 5.1<br><br>88 = Don't Know	
4.11	Does your husband want the same number of children that you want, does he want more than [illegible]	01 = Same #<br>02 = More children<br>[illegible]	

5. Infant Motor Development Milestones[4]

Read: Now I would like to ask you about (INFANT NAME).

5.1	How old is INFANT NAME?	Days<br>Months<br>88 = Don't Know	

Read: Now I would like to specifically begin with a few questions about how (INFANT NAME) is developing, and his/her ability to move.

5.2	Can (INFANT NAME) sit without any help, assistance, or support?	01 = Yes<br>02 = No<br>88 = Don't Know	
5.3	Can (INFANT NAME) stand with assistance or support?	01 = Yes<br>02 = No<br><br>77 = Yes, stands without assistance or support<br>88 = Don't Know	
5.4	Can (INFANT NAME) crawl on his/her hands and knees?	01 = Yes<br>02 = No<br>88 = Don't Know	
5.5	Can (INFANT NAME) walk with assistance or support?	01 = Yes<br>02 = No<br><br>77 = Yes, walks without assistance or support<br>88 = Don't Know	
5.6	Can (INFANT NAME) stand alone, with no assistance or support?	01 = Yes<br>02 = No<br>88 = Don't Know	
5.7	Can (INFANT NAME) walk alone, with no assistance or support?	01 = Yes<br>02 = No<br>88 = Don't Know	

[4] WHO Multicentre Growth Reference Study Group. WHO Motor Development Study: Windows of achievement for six gross motor development milestones. Acta Paediatrica Supplement 2006;450:86-95.

6. Infant Health[5]

INSTRUCTIONS: Prior to interview, check (INFANT NAME)'s hemoglobin (Hb) level recorded in Q-10.10 of the 3 Month Questionnaire. If Hb was normal (greater than or equal to 11.0 g/dL), please skip to Q-6.9. Any infant with low Hb (less than 11.0 g/dL) should have been referred to a local health clinic. Please proceed to ask Q-6.1 to Q-6.7.

6.0	**For INTERVIEWER:**<br>Did (INFANT NAME) have a Hb level lower than 11.0 g/dL?	01 = Yes<br>02 = No >> Skip to 6.9<br>88 = Don't Know	

READ: (MOTHER NAME), when we came about 6 months ago, (INFANT NAME)'s hemoglobin was 11.0 g/dL. This is very low, which suggests that he/she has low levels of iron in his/her blood and potentially iron deficiency anemia. I would like to now ask you a few questions about this.

6.1	Did you seek advice or treatment about the low hemoglobin levels in his/her blood?	01 = Yes<br>02 = No >> Skip to 6.5<br>88 = Don't Know	
6.2	What was the advice or treatment received?<br><br>***Instructions***: Please record all responses.	01 = Eat more iron-rich foods as complementary feeding begins<br>02 = Iron tablets<br>03 = Iron syrup<br>04 = Iron-fortified foods<br>05 = Consuming more diverse diet<br>06 = Including vitamin C in diet<br>07 = *Pushtika* (Sprinkles/MNP)<br><br>08 = Breastfeeding more<br>09 = Breastfeed less<br><br>10 = Deworming medication<br><br>11 = Other. Please specify:<br>________________<br><br>88 = Don't Know	
6.3	Who did you seek advice or treatment from?<br><br>***Instructions***: Please record all responses.	01 = Health Personnel / Qualified Doctor<br>02 = Nurse/Midwife/Paramedic<br><br>03 = Community Counselor<br>04 = Community Health and Nutrition Worker<br>05 = Community Health and Nutrition Mobilizer<br><br>06 = Family Welfare Visitor<br>07 = Community Skilled Birth Attendant<br>08 = MA/SACMO<br>09 = Health Assistant<br>10 = Family Welfare Assistant<br>11 = Trained TBAs<br>12 = Untrained TBA<br>13 = Unqualified Doctor<br>14 = Pharmacist/Drug seller<br><br>77 = Other. Specify: ________________<br>88 = Don't Know<br>99 = Not Applicable	

[5] Adapted from Bangladesh Demographic and Health Survey (BDHS) 2007. Additional questions about infant feeding during diarrhea, fever, cough, and other illnesses; as well as MNP added.

6.4	Where did you seek advice or treatment from?<br><br>***Instructions***: Please record all responses.	01 = Upazila Health Complex<br>02 = Family Welfare Center<br>03 = Community Clinic<br>04 = Satellite Clinic<br>05 = Home<br>06 = MTMSG<br>07 = Men's Group<br>08 = EPI Clinic<br>09 = GMP<br>10 = Pharmacy<br><br>11 = Other. Specify ___________________<br><br>88 = Don't Know	
6.5	Since you have known that (INFANT NAME) has low hemoglobin levels, have there been any changes in the types of foods that (INFANT NAME) eats?	01 = Yes<br>02 = No >> Skip to 6.7<br>88 = Don't Know	
6.6	When you started giving foods, what changes did you make in comparison with how you would have fed if (INFANT NAME) did not have low Hb?<br><br>*Instructions: Please record ALL responses.*<br><br>01 = More<br>02 = Less<br><br>99 = Not Applicable	Cereals (rice, smashed rice/gruel, bread, noodles, biscuits, or any other foods made from rice, wheat)<br><br>Vitamin A Rich Vegetables/Tubers (pumpkin, carrots, or sweet potatoes that are orange inside + other locally available vit A-rich vegetables)<br><br>White Tubers and Roots (White potatoes or foods made from tubers/roots)<br><br>Dark Leafy Green Vegetables (dark green/leafy vegetables locally available vitamin-A rich leaves, e.g. amaranth leaves etc)<br><br>Vitamin A Rich Fruits (ripe mangoes, papaya, jackfruit or other locally available vitamin A-rich fruits)<br><br>Vitamin C Rich Fruits (orange, papaya)<br><br>Other Vegetables (e.g. tomatoes, eggplant)<br><br>Other Fruits (e.g. guava, pineapple, watermelon, melon, apple, grape, banana)<br><br>Organ Meat (liver, kidney, heart, or others)<br><br>Flesh Meats (beef, lamb, goat, chicken, duck, or other birds)<br><br>Eggs<br><br>Fish (fresh/dried fish or shellfish	

		Legumes, Nuts, and Seeds (beans, peas, lentils, nuts, seeds, or foods made from these)<br><br>Milk (cow's, goat's), cheese, yogurt, or other milk products<br><br>Oil, fats, or butter added to food or used for cooking<br><br>Cookies, cake, biscuits, chips, sweets, samocha<br><br>Nutritional supplements (*Pushtika* (Sprinkles/MNP))<br><br>Other(s). Specify____________________	
6.7	Since you have known that (INFANT NAME) has very low hemoglobin levels, have there been any changes in the amount of breast milk that (INFANT NAME) drinks?	01 = Yes<br>02 = No >> Skip to 6.9<br>88 = Don't Know	
6.8	What were the changes in the amount of breast milk that (INFANT NAME) drinks? More, less, or about the same?	01 = Much Less<br>02 = Somewhat Less<br>03 = About the Same<br>04 = More<br>88 = Don't Know<br>99=Not applicable	

READ: Now I would like to ask about (INFANT NAME)'s health in general.

6.9	Since birth, has (INFANT NAME) ever received a vitamin A dose (like this/any of these)?<br><br>Show common types of capsules.	01 = Yes<br>02 = No<br>88 = Don't Know	
6.10	Has (INFANT NAME) received deworming medication in the past year?	01 = Yes<br>02 = No<br>88 = Don't Know	

READ: Now I would like to ask about (INFANT NAME)'s health just in the past two weeks.

6.11	Has (INFANT NAME) had diarrhea in the last 2 weeks?	01 = Yes<br>02 = No >> Skip to 6.22<br>88 = Don't Know	
6.12	While (INFANT NAME) had diarrhea, was he/she given breast milk?	01 = Yes<br>02 = No >> Skip to 6.14<br>88 = Don't Know	

6.13	While (INFANT NAME) had diarrhea, how much <u>breast milk</u> was he/she given to drink?<br><br>Was he/she given less than usual breast milk to drink, about the same amount, or more than usual to drink?<br><br>IF LESS, PROBE: Was he/she given much less than usual to drink or somewhat less?	01 = Much Less<br>02 = Somewhat Less<br>03 = About the Same<br>04 = More<br>88 = Don't Know<br>99=Not applicable	
6.14	While (INFANT NAME) had diarrhea, was he/she given liquids (aside from breast milk) to drink?	01 = Yes<br>02 = No >> Skip to 6.16<br>88 = Don't Know	
6.15	While (INFANT NAME) had diarrhea, how much <u>other liquids</u> (aside from breast milk) was he/she given to drink?<br><br>Was he/she given less than usual other liquids to drink, about the same amount, or more than usual to drink?<br><br>IF LESS, PROBE: Was he/she given much less than usual to drink or somewhat less?	01 = Much Less<br>02 = Somewhat Less<br>03 = About the Same<br>04 = More<br>88 = Don't Know<br>99=Not applicable	
6.16	When (INFANT NAME) had diarrhea, was he/she given less than usual to eat, about the same amount, more than usual to eat, or nothing to eat?<br><br>Instructions: ONLY ask if the infant is not exclusively breastfed. If the infant is still exclusively breastfed, mark '99'/'Not Applicable'<br><br>IF LESS, PROBE: Was he/she given much less than usual to eat or somewhat less?	01 = Much Less<br>02 = Somewhat Less<br>03 = About the Same<br>04 = More<br>05 = Nothing to Eat<br>88 = Don't Know<br>99 = Not Applicable	
6.17	When (INFANT NAME) had diarrhea, was he/she given *Pushtika* (Sprinkles/MNP) with food?	01 = Yes<br>02 = No >> Skip to 6.19<br>88 = Don't Know<br>99 = Not Applicable	
6.18	How many sachets of *Pushtika* (Sprinkles/MNP) was (INFANT NAME) given while he/she had diarrhea?	# of sachets<br><br>88 = Don't Know<br>99 = Not Applicable	
6.19	Did you seek advice or treatment when (INFANT NAME) has diarrhea?	01 = Yes<br>02 = No >> Skip to 6.22	
6.20	What was the advice or treatment received?<br><br>***Instructions***: Please record all responses.	01 = ORS<br>02 = zinc<br>03 = ORS + zinc<br>04 = Drinking more liquids (aside from breast milk)<br>05 = Drinking less liquids (aside from breast	

		milk)<br>06 = Drinking more breast milk than usual<br>07 = Drinking less breast milk than usual<br>08 = Antibiotics<br>88 = Don’t Know<br>99=Not applicable	
6.21	Where did you seek advice or treatment from?<br><br>***Instructions***: Please record all responses.	01 = Health Personnel / Qualified Doctor<br>02 = Nurse/Midwife/Paramedic<br><br>03 = Community Counselor<br>04 = Community Health and Nutrition Worker<br>05 = Community Health and Nutrition Mobilizer<br><br>06 = Family Welfare Visitor<br>07 = Community Skilled Birth Attendant<br>08 = MA/SACMO<br>09 = Health Assistant<br>10 = Family Welfare Assistant<br>11 = Trained TBA<br>12 = Untrained TBA<br>13 = Unqualified Doctor<br>14 = Pharmacist/Drug Seller<br><br>77 = Other(Specify) ________________<br><br>88 = Don’t know<br>99=Not applicable	
6.22	Has (INFANT NAME) been ill with a fever at any time in the last 2 weeks?	01 = Yes<br>02 = No<br><br>88 = Don’t Know	
6.23	Has (INFANT NAME) had an illness with a cough at any time in the last 2 weeks?	01 = Yes<br>02 = No >> Skip to 7.1<br><br>88 = Don’t Know	
6.24	When (INFANT NAME) had an illness with a cough, did he/she breathe faster than usual with short, rapid breaths or have difficulty breathing?	01 = Yes<br>02 = No >> Skip to 7.1<br>88 = Don’t Know<br>99=Not applicable	
6.25	Was the fast or difficult breathing due to a problem in the chest or to a blocked or runny nose?	01 = Chest only<br>02 = Nose only<br>03 = Both<br>77 = Other _________Specify<br>88 = Don’t Know<br>99=Not applicable	

Mother Study ID | | | | |

7. Infant Nutrition: Diet

Read: Now I would like to ask you about any foods or liquids that (INFANT NAME) ate in the last 24 hours. First, I would like to begin with liquids.

7.1	In the past 24 hours, did (INFANT NAME) drink:		
	A. Breast milk?	01 = Yes<br>02 = No<br>88 = Don't Know	
	B. Plain water?	01 = Yes<br>02 = No<br>88 = Don't Know	
	C. Sugar Water/Honey/Juice	01 = Yes<br>02 = No<br>88 = Don't Know	
	D. Commercially produced infant formula/baby formula?	01 = Yes<br>02 = No<br>88 = Don't Know	
	E. Cow's milk?	01 = Yes<br>02 = No<br>88 = Don't Know	
	F. Goat's milk?	01 = Yes<br>02 = No<br>88 = Don't Know	
	G. Yoghurt?	01 = Yes<br>02 = No<br>88 = Don't Know	
	H. Luta ("suji", dilute semolina)	01 = Yes<br>02 = No<br>88 = Don't Know	
	I. Other liquid (aside from breast milk or the liquids just previously named)?	01 = Yes<br>02 = No<br>03 = ORS<br>88 = Don't Know<br>If Yes, please specify:<br>__________<br>__________	

Mother Study ID | | | | |

Instructions:

Please COMPLETE ALL OF COLUMN FOR Q-7.2 BEFORE BEGINNING COLUMN FOR Q-7.3, and all of COLUMN Q-7.3 before COLUMN Q-7.4.

Column for Q-7.2:	Please code '01' if the infant has consumed the food mentioned in the list in past 24 hours, otherwise code '02.' Circle the food name that has been consumed.
Column for Q-7.3:	Only for each type of the food that was eaten, ask about how often (how many times per day) the food was eaten.
Column for Q-7.4:	Only for each type of food that was eaten, ask about the consistency of the food.

Read: Now I would like to ask you about any foods (INFANT NAME) had in the last 24 hours.

	Food list	*Examples*	*Q-7.2*	*Q-7.3*	*Q-7.4*
			In the last 24 hours, did (INFANT NAME) eat (FOOD)?	*How many times in the last 24 hours was (FOOD) eaten?*	*What was the consistency of _______? Was it: solid (the same as what you or other family members ate); semi-solid (with some water added); or soft (runny, diluted with water)?*
			01=Yes<br>*02=No*	*# of times/day*<br>*88 = Don't Know*	*01 = Solid food (same as other family eats)*<br>*02 = Semi-solid (some water added)*<br>*03 = Soft (dilute, water added)*<br>*04 = Water-based (e.g. soup/broth consumed, cooked with food in it)*
A.	CEREALS	Rice, smashed rice/rice gruel (suji), bread, noodles, biscuits, or any other foods made from wheat or rice, luta			*Insructions: Code LUTA = 03*<br>*SUJI = 02*
B.	VITAMIN A RICH VEGETABLES AND TUBERS	pumpkin, carrots or sweet potatoes that are orange inside + other locally available vitamin-A rich vegetables			
C.	WHITE TUBERS AND ROOTS	White potatoes or foods made from roots.			
D.	DARK GREEN LEAFY VEGETABLES	dark green/leafy vegetables locally available vitamin-A rich leaves (e.g. amaranth leaves)			
E.	OTHER VEGETABLES	other vegetables e.g. tomato, eggplant			
F.	VITAMIN A RICH FRUITS	ripe mangoes, papaya, jackfruit or other locally available vitamin A-rich fruits			
G.	VITAMIN C RICH FRUITS	Oranges, papaya			

H.	OTHER FRUITS	Other fruits (e.g. guava, pineapple, watermelon, melon, apple, grape, banana)			
I.	ORGAN MEAT (IRON RICH)	liver, kidney, heart or other organ meats			
J.	FLESH MEATS	beef, lamb, goat, chicken, duck, or other birds			
K.	EGGS	Egg			
L.	FISH	Fresh or dried fish or shellfish			
M.	LEGUMES, NUTS AND SEEDS	beans, peas, lentils, nuts, seeds or foods made from these			
N.	MILK AND MILK PRODUCTS	milk (cow's, goat's), cheese, yogurt or other milk products			
O.	OILS AND FATS	oil, fats or butter added to food or used for cooking			
P.	JUNK FOOD	cookies, cakes, biscuits, chips, sweets, samocha			
Q.	NUTRITIONAL SUPPLEMENTS	*Pushtika* (Sprinkles/MNP)			

Mother Study ID | | | | |

Read: I would now like to ask you a few questions about what (INFANT NAME) eats and drinks.

7.5	***At what age was (INFANT NAME) first given any liquids to drink, other than breast milk?***	01 = Never given breast milk<br><br>02 = Immediately (within minutes)<br>03 = Within 1 hour<br>04 = 2 hours<br>05 = 3-12 hours<br>06 = 13-24 hours	07 = 2-3 days<br>08 = 1 week<br>09 = 2 weeks<br>10 = 1 month<br>11 = 2 months<br>12 = 3 months<br>13 = 4 months	14 = 5 months<br>15 = 6 months<br>16 = 7 months<br>17 = 8 months<br>18 = 9 months<br>19 = 10 months<br><br>20 = Other. Specify: _________<br>88 = Don't Know	
7.6	***At what age was (INFANT NAME) first given water to drink?***	01 = Never given water<br><br>02 = Immediately (within minutes)<br>03 = Within 1 hour<br>04 = 2 hours<br>05 = 3-12 hours<br>06 = 13-24 hours	07 = 2-3 days<br>08 = 1 week<br>09 = 2 weeks<br>10 = 1 month<br>11 = 2 months<br>12 = 3 months<br>13 = 4 months	14 = 5 months<br>15 = 6 months<br>16 = 7 months<br>17 = 8 months<br>18 = 9 months<br>19 = 10 months<br><br>20 = Other. Specify: _________<br>88 = Don't Know	
7.7	***At what age was (INFANT NAME) first given solid (or semi-solid/soft) foods to eat?***	01 = Never given foods<br><br>02 = Immediately (within minutes)<br>03 = Within 1 hour<br>04 = 2 hours<br>05 = 3-12 hours<br>06 = 13-24 hours	07 = 2-3 days<br>08 = 1 week<br>09 = 2 weeks<br>10 = 1 month<br>11 = 2 months<br>12 = 3 months<br>13 = 4 months	14 = 5 months<br>15 = 6 months<br>16 = 7 months<br>17 = 8 months<br>18 = 9 months<br>19 = 10 months<br><br>20 = Other. Specify: _________<br>88 = Don't Know	

READ: Now I would like to ask you about any Pushtika (Sprinkles/MNP) nutritional supplements that (INFANT NAME) eats or has eaten.

7.8	***Have you used Sprinkles/MNP to feed (INFANT NAME)?***		01 = Yes<br>02 = No >> Skip to 8.1<br><br>88 = Don't Know	
7.9	***How many TIMES PER WEEK has Pushtika (Sprinkles/MNP) been added to (INFANT NAME)'s foods?***	01 = Once / week<br>02 = 2-3 times/week<br>03 = 4-5 times/week<br>04 = 6-7 times/week<br>05 = >7 times per week	77 = Other. Specify<br>_______________________<br>88 = Don't Know<br>99 = Not Applicable	
7.10	***Is (INFANT NAME) ever given Pushtika (Sprinkles/MNP) more than once per day?***		01 = Yes<br>02 = No >>Skip to 7.12<br><br>88 = Don't Know	
7.11	***How many TIMES PER DAY has Pushtika (Sprinkles/MNP) been added to (INFANT NAME)'s foods?***	01 = >=3 times/day<br>02 = 2 times/day<br>03 = 1 time/days	77 = Other. Specify<br>_______________________<br>88 = Don't Know<br>99 = Not Applicable	
7.12	***How many sachets of Pushtika did you usually use in one day?***	01 = < 1/day<br>02 = 1/day<br>03 = between 1 and 2/day<br>04 = 2/day<br>05 = > 2/day	77 = Other. Specify<br>_______________________<br>88 = Don't Know<br>99 = Not Applicable	

READ: Now I would like to ask some questions about how you fed Pushtika.

	Question	Box 1	Box 2
7.13	Which of the following did you use to feed (INFANT NAME) when giving *Pushtika?*<br><br>*Instructions*: Read responses. Check all that apply.	**7.13 A**<br>01 = Plate<br>02 = Bati<br>03 = Feeder bottle<br><br>77 = Other ___________<br>88 = Do not remember	**7.13 B**<br>01 = Plate<br>02 = Bati<br>03 = Feeder bottle<br><br>77 = Other ___________<br>88 = Do not remember
7.14	When giving *Pushtika to* (INFANT NAME), which of the following kinds of main food did you usually use to mix it?<br><br>*Instructions*: Read responses. If answer is 'c' (rice) proceed to question 7.15, otherwise skip to question 7.23.	**7.14 A**<br>01 = Sugi<br>02 = Luta<br>03 = Mashed rice<br>04 = Rice<br><br>77 = Other _____________<br>88 = Do not remember	**7.14 B**<br>01 = Sugi<br>02 = Luta<br>03 = Mashed rice<br>04 = Rice<br><br>77 = Other _____________<br>88 = Do not remember
7.15	How much [response to question 7.14] did you usually offer for (INFANT NAME) when you gave *Pushtika* from the [first/second] box you received?	**7.15 A**<br>01 = Less than ½ bowl (bati)<br>02 = ½ to 1 bowl (bati)<br>03 = ≥ 1 bowl (bati)<br><br>77 = Other _____________<br>88 = Do not remember	**7.15 B**<br>01 = Less than ½ bowl (bati)<br>02 = ½ to 1 bowl (bati)<br>03 = ≥ 1 bowl (bati)<br><br>77 = Other _____________<br>88 = Do not remember
7.16	How much [response to question 7.14] did you usually feed to (INFANT NAME) when you gave *Pushtika* from the [first/second] box you received?	**7.16 A**<br>01 = Less than ½ bowl (bati)<br>02 = ½ to 1 bowl (bati)<br>03 = ≥ 1 bowl (bati)<br><br>77 = Other _____________<br>88 = Do not remember	**7.16 B**<br>01 = Less than ½ bowl (bati)<br>02 = ½ to 1 bowl (bati)<br>03 = ≥ 1 bowl (bati)<br><br>77 = Other _____________<br>88 = Do not remember
7.17	Did you ever mix any of the family food in the meal with *Pushtika* from the [first/second] box?<br><br>*Instructions*: If 'yes' proceed to question 7.18, otherwise skip to question 7.23 or 7.26.	**7.17 A**<br>01 = Yes<br>02 = No<br><br>77 = Other _____________<br>88 = Do not remember	**7.17 B**<br>01 = Yes<br>02 = No<br><br>77 = Other _____________<br>88 = Do not remember
7.18	When feeding *Pushtika* from the [first/second] box, how many different foods did you usually mix together with [response from question 7.14]?	**7.18 A**<br>00 = 0<br>01 = 1<br>02 = 2<br>03 = 3<br>04 = 4<br>05 = 5+<br><br>77 = Other _____________<br>88 = Do not remember	**7.18 B**<br>00 = 0<br>01 = 1<br>02 = 2<br>03 = 3<br>04 = 4<br>05 = 5+<br><br>77 = Other _____________<br>88 = Do not remember
7.19	When feeding *Pushtika* from the [first/second] box, which of the following kinds of foods did you usually mix with [response from question 7.14]?	**7.19 A**<br>01 = Vegetables<br>02 = Fish<br>03 = Meat (beef, chicken, mutton)<br><br>77 = Other _____________<br>88 = Do not remember	**7.19 B**<br>01 = Vegetables<br>02 = Fish<br>03 = Meat (beef, chicken, mutton)<br><br>77 = Other _____________<br>88 = Do not remember
7.20	When giving *Pushtika* from the [first/second] box, how much of the foods did usually you mix in?	**7.20 A**<br>01 = A pinch<br>02 = A "finger-grab"<br>03 = A small spoonful<br>04 = A large spoonful<br><br>77 = Other _____________<br>88 = Do not remember	**7.20 B**<br>01 = A pinch<br>02 = A "finger-grab"<br>03 = A small spoonful<br>04 = A large spoonful<br><br>77 = Other _____________<br>88 = Do not remember
7.21	When feeding *Pushtika* from the [first/second] box, did you ever add oil or fry any of the foods that you mixed with [response from question7.14]?<br><br>*Instructions*: If 'Yes' for either, then proceed to question 7.22 for either box, otherwise skip to question 7.23.	**7.21 A**<br>01 = Yes<br>02 = No<br><br>77 = Other _____________<br>88 = Do not remember	**7.21 B**<br>01 = Yes<br>02 = No<br><br>77 = Other _____________<br>88 = Do not remember

	Question	Box 1	Box 2
7.22	How often did you add oil or fry foods that you mixed in?	**7.22 A**<br>01 = Every day<br>02 = 4-6 days/week<br>03 = 2-3 days/week<br>04 = 1 day/week<br>05 = Less often than 1 day/week<br><br>77 = Other _____________<br>88 = Do not remember	**7.22 B**<br>01 = Every day<br>02 = 4-6 days/week<br>03 = 2-3 days/week<br>04 = 1 day/week<br>05 = Less often than 1 day/week<br><br>77 = Other _____________<br>88 = Do not remember
7.23	Did you receive a second box of *Pushtika*?<br><br>*Instructions*: If 'Yes' proceed to question 7.24. If 'No' skip to question 7.25.	**7.23**<br>01 = Yes<br>02 = No<br><br>77 = Other _____________<br>88 = Do not remember	
7.24	How many sachets were remaining from the first box of *Pushtika* when you received the second box?	____ (Record number.)<br><br>88 = Do not remember	
7.25	How many sachets were left over from the box of *Pushtika* you received? [END OF QUESTIONS IF ONLY RECEIVED ONE BOX].	____ (Record number.)<br><br>88 = Do not remember	
7.26	How many sachets are left over altogether from the first and second box of *Pushtika* that you received?		____ (Record number.)<br><br>88 = Do not remember.

8. Intentions for Introducing Foods

READ: Now I would like to ask you some questions about your plans to feed your baby. Please choose the answer that most clearly matches your opinion considering both your plans and the likelihood that you will carry out those plans.

8.1	***How did you know when it is time to feed (INFANT NAME) foods in addition to breast milk?***	01 = Infant restless when breastfeeding<br>02 = Infant distracted when breastfeeding<br>03 = Infant shows interest when others are eating solid foods, or drinking from cups<br>04 = Infant makes gestures indicating interest in being fed other foods<br>05 = Disappearance of tongue thrusting reflex/motion<br>06 = Infant not satisfied with only breast milk<br><br>07 = Other. Specify ___________________<br>88 = Don't know	
8.2 A	***In the first few days after you began giving (INFANT NAME) other foods besides breast milk, how many times a day did you give (INFANT NAME) foods?***	Times a Day<br><br>88=Don't know<br><br>99 = Not applicable	
8.2 B	***In the first month after you began giving (INFANT NAME) other foods besides breast milk, how many times a day did you give (INFANT NAME) foods?***	Times a Day<br><br>88=Don't know<br><br>99 = Not applicable	
8.2 C	***In the first three months after you began giving (INFANT NAME) other foods besides breast milk, how many times a day did you give (INFANT NAME) foods?***	Times a Day<br><br>88=Don't know<br><br>99 = Not applicable	
8.3	***In the next 6 months from today, how many times a day, do you plan to give (INFANT NAME) foods?***	Times a Day<br><br>88=Don't know<br><br>99 = Not Applicable (Still EBF)	
8.4	***When you first began giving (INFANT NAME) other foods, what types of foods did you first give to (him/her) in the first week he/she eats foods?***<br><br>01 = Yes<br>02 = No	Luta<br><br>Cereals (rice, smashed rice/gruel, bread, noodles, biscuits, or any other foods made from rice, wheat)<br><br>Vitamin A Rich Vegetables/Tubers (pumpkin, carrots, or sweet potatoes that are orange inside + other locally available vit A-rich vegetables)<br><br>White Tubers and Roots (White potatoes or foods made from tubers/roots)<br><br>Dark Leafy Green Vegetables (dark green/leafy vegetables locally available vitamin-A rich leaves, e.g. amaranth leaves etc)<br><br>Vitamin A Rich Fruits (ripe mangoes, papaya, jackfruit or other locally available vitamin A-rich fruits)<br><br>Vitamin C Rich Fruits (orange, papaya)	

		Other Vegetables (e.g. tomatoes, eggplant)	
		Other Fruits (e.g. guava, pineapple, watermelon, melon, orange, apple, grape, banana)	
		Organ Meat (liver, kidney, heart, or others)	
		Flesh Meats (beef, lamb, goat, chicken, duck, or other birds)	
		Eggs	
		Fish (fresh/dried fish or shellfish	
		Legumes, Nuts, and Seeds (beans, peas, lentils, nuts, seeds, or foods made from these)	
		Milk (cow's, goat's), cheese, yogurt, or other milk products	
		Oil, fats, or butter added to food or used for cooking	
		Cookies, cake, biscuits, chips, sweets, samocha	
		Nutritional supplements (*Pushtika* (Sprinkles/MNP))	
		Other(s). Specify____________________<br>_________________________________	
8.5	***When (INFANT NAME) is 1 year old, what additional types of foods do you plan to introduce to his/her diet?***<br><br>01 = Yes<br>02 = No<br><br>99=Not Applicable (Still EBF)	Luta	
		Cereals (bread, noodles, biscuits, or any other foods made from rice, wheat)	
		Vitamin A Rich Vegetables and Tubers (pumpkin, carrots, or sweet potatoes that are orange inside + other locally available vitamin A-rich vegetables)	
		White Tubers and Roots (White potatoes or foods made from roots)	
		Dark Leafy Green Vegetables (dark green/leafy vegetables locally available vitamin-A rich leaves such as amaranth leaves etc)	
		Vitamin A Rich Fruits (ripe mangoes, papaya, jackfruit or other locally available vitamin A-rich fruits)	
		Vitamin C Rich Fruits (orange, papaya)	
		Other Vegetables (e.g. tomatoes, eggplant)	

		Other Fruits (e.g. guava, pineapple, watermelon, melon, orange, apple, grape, banana)	
		Organ Meat (iron rich)	
		Flesh Meats (beef, lamb, goat, chicken, duck, or other birds)	
		Eggs	
		Fish (fresh/dried fish or shellfish	
		Legumes, Nuts, and Seeds (beans, peas, lentils, nuts, seeds, or foods made from these)	
		Milk (cow's, goat's), cheese, yogurt, or other milk products	
		Oil, fats, or butter added to food or used for cooking	
		Nutritional supplements (*Pushtika* (Sprinkles/MNP))	
		Other(s). Specify_____________________ ___________________________________	
8.6	***When (INFANT NAME) is 1 year old, how do you plan to prepare the foods that he/she eats?***<br><br>***Instructions: Please READ all choices, and record ALL responses.***<br><br>***01 = Yes***<br>***02 = No***<br><br>***88 = Don't Know***	Same food as for family	
		Different food than family	
		Watered down food	
		Pureed	
		Mashed	
		Semi-solid	
		Other (Specify)<br>---<br>---	

8.7	***Instructions: In this section, ONLY ask either questions A or B. Based on respondent's answers in Section 8, decide whether the infant is:***<br>***A) Currently still breastfed (if 7.1-A only has 01 (YES) for breast milk)***<br>***B) Not breastfed (if 02 (NO) is marked for breast milk in 7.1-A)***	**A = 01**	**B = 02**	

	A –STILL BREASTFED	B – NOT BREASTFED		
8.8	***At what age do you plan to STOP giving (INFANT NAME) breast milk?***<br><br>***Instructions:*** *If needed, clarify: When he/she is how many months old?*	***At what age did you STOP giving (INFANT NAME) breast milk?***<br><br>***Instructions:*** *If needed, clarify: When he/she was how many months old?*	# of Months<br><br>00 if <01 Month<br><br>01 = When he/she loses interest<br>02 = Other. Specify<br>______________________<br><br>88=Don't Know	
8.9	***When did you decide at what age you are going to STOP giving (INFANT NAME) breast milk?***	***When did you decide at what age you were going to STOP giving (INFANT NAME) breast milk?***	01 = Before you became pregnant<br>02 = During pregnancy<br>03 = After the baby's birth<br>04 = During the first three months after birth<br>05 = Between 3 months and 9 months after birth<br>06 = Did not make any decision<br><br>88=Don't know<br><br>99= Not Applicable (Not BF)	

Read: Now I would like to ask you a few questions about nutritional supplements, and any plans to feed (INFANT NAME) nutritional supplements.

8.10	***Have you heard of Pushtika (Sprinkles/MNP) or multiple-micronutrient powders?***	01 = Yes<br>02 = No >> Skip to 8.15<br><br>88 = Don't know >> Skip to 8.15	
8.11	***When (INFANT NAME) is 1 year old, do you plan to add any Pushtika (Sprinkles/MNP) to his/her foods?***	01 = Yes<br>02 = No >> Skip to 8.15<br><br>03 = Already give *Pushtika* (Sprinkles/MNP)<br><br>88 = Don't know	
8.12	***How many times/week do you plan to give (INFANT NAME) Pushtika (Sprinkles/MNP) with his/her foods?***	# of times/week	
8.13	***Why do you plan to give (INFANT NAME) Pushtika (Sprinkles/MNP)?***<br><br>***Please record ALL responses.***<br><br>***Instructions: If 8.15 is answered, SKIP TO 8.17***	01 = Good for baby<br>02 = Easy to feed *Pushtika* (Sprinkles/MNP)<br>03 = Reasonable price<br>04 = Recommended by friends<br>05 = Recommended by doctor/health professional<br>06 = Others also use *Pushtika* (Sprinkles/MNP)<br>07 = To avoid anemia<br>08 = To provide iron<br><br>09 = Other(s). Please specify:<br>______________________<br>______________________	
8.14	***Why do you NOT plan to give (INFANT NAME) Pushtika (Sprinkles/MNP)?***<br><br>***Please record ALL responses.***	01 = Difficult to find in stores<br>02 = Too expensive<br>03 = No need to<br>04 = Doctor never recommended<br>05 = My family does not think necessary<br>06 = I don't think it is necessary<br>07 = My friends do not think it is necessary<br><br>08 = Other(s). Please specify:	

		______________________________<br>______________________________	

READ: Now I would like to ask you a few more questions about how you plan to feed (INFANT NAME) when he/she has diarrhea. After each of the following questions, I will read out options. Please either agree (by saying YES) or disagree (by saying NO) with the statement.

8.15	***When (INFANT NAME) has diarrhea or becomes sick, how do you plan to feed him/her?***<br><br>*INSTRUCTIONS:* ***Please read ALL options, and*** *record all responses.*<br><br>*PROBE: To clarify between different choices (e.g. less or more than usual?)*<br><br>01 = Yes<br>02 = No<br>99 = Not Applicable (If no response)	Continue breastfeeding >> If No, Skip next two choices (BF less or more than usual)<br>Breastfeed less than usual<br>Breastfeed more than usual<br>Give less foods than usual<br>Give as much foods as usual<br>Give more food than usual<br>Give less liquids than usual<br>Give as much liquids as usual<br>Give more liquids than usual<br>Give syrups<br>Give traditional medicine<br>Give treated water<br>Give carrot juice or rice water<br>Give Zinc<br>ORS<br>Other [Specify]-------------------------------	
8.16	***After (INFANT NAME) recovers from diarrhea or sickness, how do you plan to feed him/her?***<br><br>*INSTRUCTIONS:* ***Please read ALL options, and*** *record all responses.*<br><br>*PROBE: To clarify between different choices (e.g. less or more than usual?)*<br><br>01 = Yes<br>02 = No<br>99 = Not Applicable (If no response)	Continue breastfeeding >> If No, Skip next two choices (BF less or more than usual)<br>Breastfeed less than usual<br>Breastfeed more than usual<br>Give less foods than usual<br>Give as much foods as usual<br>Give more food than usual<br>Give less liquids than usual<br>Give as much liquids as usual<br>Give more liquids than usual<br>Give syrups<br>Give traditional medicine<br>Give treated water	

		Give carrot juice or rice water	
		Give Zinc	
		ORS	
		Other [Specify]-------------------------------	

Read: I would now like to ask you a few questions about HOW you plan to feed (INFANT NAME).

8.17	***Imagine that you are having trouble feeding (INFANT NAME). What are ways that you plan to try, in order to feed him/her?***<br><br>*INSTRUCTIONS: Please record all that apply*<br>01= Yes<br>99=Not applicable (if not responded)	Active encouragement	
		Giving infant his/her own plate	
		Force feeding	
		Introducing new foods one at a time	
		Talk to child while feeding	
		Maintaining eye-to-eye contact	
		Minimize distractions during meals	
		Include a variety of foods slowly	
		Include nutritional supplements	
		Play (as encouragement)	
		Try different foods	
		Add sugar	
		Dilute with water	
		Contact, seek advice from CC, CHNW, CCNM	
		Other. Specify __________________	

9. Knowledge on Infant Feeding & Development at Age One

READ: Now I would like to ask you some general questions regarding feeding your baby foods. After each of the following statements, I will read out options. Please either agree (by saying YES) or disagree (by saying NO) with the statement.

Instructions: Read ALL the options.

9.1	***Until what age is it recommended that a baby continues to drink breast milk?***	Record # of months<br><br>00= Less than 01 month after baby's birth<br><br>88=Don't know<br><br>99= Not Applicable (if no response)	
9.2	***How long after birth is it recommended that a baby begin to eat foods, aside from breast milk?***	00 = Less than 01 month after baby's birth<br>01 = At 1 month 07 = At 7 months<br>02 = At 2 months 08 = At 8 months<br>03 = At 3 months 09 = At 9 months<br>04 = At 4 months 10 = At 10 months<br>05 = At 5 months 11 = At 11 months<br>06 = At 6 months 12 = At 12 months<br><br>13 = >12 months (please specify)<br><br>88=Don't know<br><br>99= Not Applicable (if no response)	

9.3	***When feeding a meal to a child who is 1 year old, what are recommended types of foods to include in a single meal?***<br><br>*INSTRUCTIONS: Please record all that apply*<br><br>01 = Yes<br>02 = No<br>99 = Not Applicable (If no response)	Cereals (rice, wheat, jawar, millets, hotchpotch)	
		Legumes (lentils, pulses, beans etc)	
		Foods from animals (egg, meat, fish)	
		Milk and milk products	
		Oil or fat	
		Sugar	
		Vegetables	
		Fruits	
		Nutritional supplements (*Pushtika* (Sprinkles/MNP))	
		"Luta" (semolina, or "suji" in a watery suspension)	
		Other (Specify)	

Read: Now I would like to ask you about general knowledge on how often foods should be fed to infants when they first begin to eat foods besides breast milk.

9.4	***In the first few days after an infant begins to eat other foods besides breast milk, how many times a day is it recommended for him/her to eat?***	Times a Day<br><br>88=Don't know<br>99 = Not applicable	
9.5	***At 1 month, after an infant begins to eat other foods besides breast milk, how many times a day is it recommended for him/her***	Times a Day<br><br>88=Don't know	

9.5	***to eat?***	99 = Not applicable	
9.6	***At 3 months, after an infant begins to eat other foods besides breast milk, how many times a day is it recommended for him/her to eat?***	Times a Day<br><br>88=Don't know<br>99 = Not applicable	

READ: Now I would like to ask you a few more general knowledge questions about feeding your baby foods, in addition to breast milk. For these statements, I will not be reading aloud options.

9.7	***When an infant is 1 year old, what are the recommended ways that his/her food be prepared?***<br><br>*INSTRUCTIONS: Please record all that apply*<br><br>01 = Yes<br>99 = Not Applicable (If no response)	Same food as for family<br>Different food than family<br>Watered down food<br>Pureed<br>Mashed<br>Semi-solid<br><br>Other (Specify)<br><br>---<br><br>---	

9.8	***Imagine there is a mother who is having trouble beginning to introduce foods to her infant. What are the recommended ways a mother can try to feed her infant foods, aside from breast milk?***<br><br>*INSTRUCTIONS: Please record all that apply*<br>01= Yes<br>99=Not applicable (if not responded)	Active encouragement<br>Giving infant his/her own plate<br>Force feeding<br>Introducing new foods one at a time<br>Talk to child while feeding<br>Maintaining eye-to-eye contact<br>Minimize distractions during meals<br>Include a variety of foods slowly<br>Include nutritional supplements<br>Play (as encouragement)<br>Other. Specify __________________	
9.9	***How often is it recommended for an infant's hands to be washed when he/she is eating foods?***<br><br>*INSTRUCTIONS; Please read all options.*	01 = Never<br>02 = Occasionally<br>03 = Sometimes<br>04 = Often<br>05 = Always<br><br>88 = Don't Know	

9.10	***How often is it recommended for a mother to wash her hands before preparing foods for her infant?***<br><br>*INSTRUCTIONS; Please read all options.*	01 = Never<br>02 = Occasionally<br>03 = Sometimes<br>04 = Often<br>05 = Always<br><br>88 = Don't Know	
9.11	***How often is it recommended for a mother to wash her hands before feeding her infant foods?***<br><br>*INSTRUCTIONS; Please read all options.*	01 = Never<br>02 = Occasionally<br>03 = Sometimes<br>04 = Often<br>05 = Always<br><br>88 = Don't Know	

READ: Now I would like to ask you a few more general knowledge questions about feeding your baby foods if he/she has diarrhea. After each of the following questions, I will read out options. Please either agree (by saying YES) or disagree (by saying NO) with the statement.

9.12	***What is recommended for a mother to do when her child has diarrhea?***<br><br>*INSTRUCTIONS:* ***Please read ALL options, and*** *record all responses.*<br><br>*PROBE: To clarify between different choices (e.g. less or more than usual?)*<br><br>01 = Yes<br>02 = No<br>99 = Not Applicable (If no response)	Continue breastfeeding >> If No, Skip next two choices (BF less or more than usual)<br><br>Breastfeed less than usual<br><br>Breastfeed more than usual<br><br>Give less foods than usual<br><br>Give as much foods as usual<br><br>Give more food than usual<br><br>Give less liquids than usual<br><br>Give as much liquids as usual<br><br>Give more liquids than usual<br><br>Give syrups<br><br>Give traditional medicine<br><br>Give treated water<br><br>Give carrot juice or rice water<br><br>Give Zinc<br><br>ORS<br><br>Other [Specify]-------------------------------	
9.13	***What is recommended for a mother to do (in relation to feeding) AFTER her child has recovered from diarrhea or another illness?***	Continue breastfeeding >> If No, Skip next two choices (BF less or more than usual)<br><br>Breastfeed less than usual	

9.13	*INSTRUCTIONS:* ***Please read ALL options, and*** *record all responses.*<br><br>*PROBE: To clarify between different choices (e.g. less or more than usual?)*<br><br>01 = Yes<br>02 = No<br>99 = Not Applicable (If no response)	Breastfeed more than usual<br>Give less foods than usual<br>Give as much foods as usual<br>Give more food than usual<br>Give more liquids than usual<br>Give nutritious food<br>Drug/vitamin<br>Other [Specify]--------------------------------	

Read: Now I would like to ask you a few questions about how Pushtika (Sprinkles/MNP) should be consumed.

9.14	***How many sachets of Pushtika (Sprinkles/MNP) is it recommended for a child to consume every day?***	01 = < 1/day<br>02 = 1/day<br>03 = between 1 and 2/day<br>04 = 2/day<br>05 = > 2/day	77 = Other. Specify<br>________________________<br>88 = Don't Know<br>99 = Not Applicable	
9.15	***How is it recommended that a mother prepares Pushtika (Sprinkles/MNP) for his/her child to eat?***	01 = Mixed with moderately warm food immediately before consumption<br>02 = Cooked with food<br>03 = Mixed with child's drink<br>04 = Mixed with child's semi-liquid food that was already cooked<br>05 = Mixed in hot food<br>06 = Mixed with water<br>07= Did not receive *Pushtika* (Sprinkles/MNP)	77 = Other. Specify<br>________________________<br>88 = Don't Know<br>99 = Not Applicable	

Read: Now I would like to ask you a few questions about where you find out information about how to feed (INFANT NAME).

9.16	Who provides you information on breastfeeding?<br><br>*Instructions: Please record all responses. After respondent finishes talking, probe "Anyone else?" until all answers are recorded.*<br><br>01 = Yes<br>99 = Not Applicable (If no response)	Mother<br>Mother in law<br>Husband<br>Elder sister<br>Sister-in-law<br>Health Assistant<br>Family Welfare Assistant<br>MTMSG facilitator<br>Community Counselor<br>Community health and nutrition workers	

		Community health and nutrition mobilizer<br>Neighbor<br>Friend<br>Other. Specify<br>____________________<br>____________________<br>Don't Know	
9.17	Who provides you information on complementary feeding, or feeding any foods or liquids aside from breast milk?<br><br>*Instructions: Please record all responses. After respondent finishes talking, probe "Anyone else?" until all answers are recorded.*<br><br>01 = Yes<br>99 = Not Applicable (If no response)	Mother<br>Mother in law<br>Husband<br>Elder sister<br>Sister-in-law<br>Health Assistant<br>Family Welfare Assistant<br>MTMSG facilitator<br>Community Counselor<br>Community health and nutrition workers<br>Community health and nutrition mobilizer<br>Neighbor<br>Friend<br>Other. Specify<br>____________________<br>____________________<br>Don't Know	
9.18	Who provides you information on *Pushtika* (Sprinkles/MNP)?<br><br>*Instructions: Please record all responses. After respondent finishes talking, probe "Anyone else?" until all answers are recorded.*<br><br>01 = Yes<br>99 = Not Applicable (If no response)	Mother<br>Mother in law<br>Husband<br>Elder sister<br>Sister-in-law<br>Health Assistant<br>Family Welfare Assistant<br>MTMSG facilitator<br>Community Counselor	

		Community health and nutrition workers<br>Community health and nutrition mobilizer<br>Neighbor<br>Friend<br>Other. Specify<br>__________<br>__________<br>- Don't Know	
9.19	Where do you gain knowledge about breastfeeding?<br><br>*Instructions: Please record all responses. Instructions: Please record all responses. After respondent finishes talking, probe "Any other places?" until all answers are recorded.*<br><br>01 = Yes<br>99 = Not Applicable (If no response)	- Counseling Sessions/Visits<br>- MTMSG<br>- Group education<br>- EPI/GMP<br>- Satellite clinic<br>- Community clinic<br>- Other. Specify<br>__________<br>__________<br>- Don't Know	
9.20	Where do you gain knowledge about complementary feeding, or feeding any foods or liquids aside from breast milk?<br><br>*Instructions: Please record all responses. After respondent finishes talking, probe "Any other places?" until all answers are recorded.*<br><br>01 = Yes<br>99 = Not Applicable (If no response)	- Counseling Sessions/Visits<br>- MTMSG<br>- Group education<br>- EPI/GMP<br>- Satellite clinic<br>- Community clinic<br>- Other. Specify<br>__________<br>__________<br>- Don't Know	
9.21	Where do you gain knowledge about *Pushtika* (Sprinkles/MNP)?<br><br>*Instructions: Please record all responses. After respondent finishes talking, probe "Any other places?" until all answers are recorded.*<br><br>01 = Yes	- Counseling Sessions/Visits<br>- MTMSG<br>- Group education	

9.21	99 = Not Applicable (If no response)	- EPI/GMP<br>- Satellite clinic<br>- Community clinic<br>- Other. Specify<br>______<br>______<br>- Don't Know	

Mother Study ID | | | | |

10. Breastfeeding and Complementary Feeding Attitudes

READ: Now I would like to ask you about your perspectives regarding feeding infants foods in addition to breast milk. Please consider the following statements about feeding your baby, (INFANT NAME). After I read each statement, please tell me whether you strongly disagree, disagree, agree, strongly agree with, or are neutral about the statement.

		**Strongly Agree 1**	**Agree 2**	**Neutral 3**	**Disagree 4**	**Strongly Disagree 5**
10.1	***I feel that I know how to successfully feed (INFANT NAME) soft and solid foods.***	1	2	3	4	5
10.2	***I am able to provide enough breast milk for my baby right now.***	1	2	3	4	5
10.3	***I will be able to provide enough breast milk for my baby when he/she is 1 year old***	1	2	3	4	5
10.4	***I am always able to provide enough breast milk for my baby right now.***	1	2	3	4	5
10.5	***I will always able to provide enough breast milk for my baby when he/she is 1 year old.***	1	2	3	4	5
10.6	***I am confident that I provide enough nutrients (both breast milk and foods) to (INFANT NAME) so that he/she is healthy and well-nourished.***	1	2	3	4	5
10.7	***I am confident that my family is able to provide (INFANT NAME) with foods so that he/she is well-nourished.***	1	2	3	4	5
10.8	***I have enough time in my day to make sure that (INFANT NAME) is properly fed.***	1	2	3	4	5
10.9	***Feeding (INFANT NAME) takes too much time in my day.***	1	2	3	4	5
10.10	***Ensuring that (INFANT NAME) is provided enough nutrients through breast milk and foods makes it difficult for me to also work.***	1	2	3	4	5
10.11	***Ensuring that (INFANT NAME) is provided enough nutrients through breast milk and foods makes it difficult for me to also finish all other household responsibilities.***	1	2	3	4	5
10.12	***I know how to recognize if my infant is hungry and full when I am feeding him/her breast milk and foods.***	1	2	3	4	5
10.13	***I am able to minimize distractions while my infant eating foods.***	1	2	3	4	5
10.14	***I am confident I can experiment with different food types and textures to find foods my infant will eat.***	1	2	3	4	5
10.15	***I am able to overcome any trouble with feeding my infant food.***	1	2	3	4	5
10.16	***I am confident I can find ways to encourage my infant to eat on his/her own.***	1	2	3	4	5
10.17	***I am determined to continue to breastfeed my infant while he/she begins to eat foods.***	1	2	3	4	5
10.18	***I am able to prepare and feed my infant foods in a clean, safe, sanitary environment.***	1	2	3	4	5
10.19	***I am able to feed (INFANT NAME) Pushtika (Sprinkles/MNP) with his/her food.***	1	2	3	4	5
10.20	***It is important that (INFANT NAME) slowly begins to eat more solid foods when he/she is ready.***	1	2	3	4	5
10.21	***I am able to obtain (or get) nutritional supplements (such as Pushtika (Sprinkles/MNP)).***	1	2	3	4	5
10.22	***I have someone who I can talk to about any problems or challenges I have with feeding foods to my baby.***	1	2	3	4	5
10.23	***I have someone who is supportive of my feeding foods to my baby when he/she is 1 year old.***<br><br>Instructions: If "Disagree" or "Strongly Disagree" marked >> Skip to 11.11	1	2	3	4	5

10.24	***Who is supportive of your feeding foods to your baby?***<br><br>Instructions: Please mark ALL responses.	01 = Husband<br>02 = Mother<br>03 = Mother-in-law<br>04 = Sister<br>07=Nobody<br>77 = Other. Specify:	

10.24	***Who is supportive of your feeding foods to your baby?***<br><br>Instructions: Please mark ALL responses.	05 = Sister-in-law<br>06 = Friend<br><br>99 = Not Applicable (No longer breastfed)	
10.25	***How would you rate your overall experience with feeding (INFANT NAME) breast milk and foods, on a scale from 1 to 5***. 1 being 'very bad, will never again simultaneously breastfeed and feed foods to future children', and 5 being 'very good, will continue to simultaneously breastfeed and feed foods to future children.'	Very Bad 1 / Bad 2 / So-So 3 / Good 4 / Very Good 5	

READ: Now I would like to ask you about who makes decisions about breastfeeding (INFANT NAME).

10.26	***Who makes (or made) decisions about up to what age (INFANT NAME) will be (or was) breastfed?***<br><br>Instructions: Please mark ALL responses.	***01 = Mother (interviewee)***<br>***02 = Father***<br>***03 = Mother's mother***<br>***04 = Mother-in-law***<br>***05 = Sister***<br>***06 = Sister-in-law***<br>***99=Not applicable***<br>***77 = Other. Specify_________________***	

READ: Now for each of the following individuals, please indicate what they believe is the best way to feed your child at 1 year (12 months) of age.

Please choose from the following five options:
When my baby is 1 year old, he/she should:

1) ***Receive only breast milk***
2) ***Receive foods without Pushtika (Sprinkles/MNP) once a day in addition to breast milk***
3) ***Receive food with Pushtika (Sprinkles/MNP) once a day in addition to breast milk***
4) ***Receive only foods without Pushtika (Sprinkles/MNP) once a day***
5) ***Receive only food with Pushtika (Sprinkles/MNP) once a day***

Please let me know if you would like me to repeat the five options at any point.

***INSTRUCTIONS*:**
If respondent does not know what the person thinks → ***mark "8" as response***
If father/mother/mother-in-law died or received no advice from doctor → ***mark "9" as the response***

10.27	***My husband thinks I should***	1	2	3	4	5	8	9
10.28	***My mother thinks I should***	1	2	3	4	5	8	9
10.29	***My mother-in-law thinks I should***	1	2	3	4	5	8	9
10.30	***My sister thinks I should***	1	2	3	4	5	8	9
10.31	***My doctor thinks I should***	1	2	3	4	5	8	9

READ: Now I would like to ask you about you and people in your family or community.<br><br>How much do you agree with the following statements?6 Please tell me whether you you agree, disagree, or are unsure.<br><br>***Since you delivered your new baby…***			
10.32	***… I have someone to help me if I am sick or need to rest***	01 = Agree<br>02 = Disagree<br>03 = Unsure<br>99=Not applicable<br>77 = Other.<br>Specify_________________	☐☐
10.33	***… I have someone to take me to the clinic or doctor's office***	01 = Agree<br>02 = Disagree<br>03 = Unsure<br>99=Not applicable<br>77 = Other.<br>Specify_________________	☐☐
10.34	***… I have someone to talk with about my problems***	01 = Agree<br>02 = Disagree<br>03 = Unsure<br>99=Not applicable<br>77 = Other.<br>Specify_________________	☐☐
10.35	***… I have someone to help me if I am tired and feeling frustrated with my new baby***	01 = Agree<br>02 = Disagree<br>03 = Unsure<br>99=Not applicable<br>77 = Other.<br>Specify_________________	☐☐

READ: Now I would like to ask you 2 additional questions, which are still about you and people in your family or community. Again, I will first read a statement. Please tell me whether you you agree, disagree, strongly agree, strongly disagree, or are unsure. 7			
10.36	***In general, I can trust the majority of people in my community.***	01 = Strongly Agree<br>02 = Agree<br>03 = Unsure<br>04 = Disagree<br>05 = Strongly Disagree<br>99=Not applicable<br>77 = Other.<br>Specify_________________	☐☐
10.37	***I feel as though I am a part of this community.***	01 = Strongly Agree<br>02 = Agree<br>03 = Unsure<br>04 = Disagree<br>05 = Strongly Disagree<br>99=Not applicable<br>77 = Other.<br>Specify_________________	☐☐

[6] Adapted from Pregnancy Risk Assessment Monitoring System and
Lippman et al. (2009) Social-Environmental Factors and Protective Sexual Behavior among Sex Workers: The *Encontros* Intervention in Brazil. Amer J Public Health, 99(11), 1-11.

[7] Adapted from Lippman et al. (2009) Social-Environmental Factors and Protective Sexual Behavior among Sex Workers: The *Encontros* Intervention in Brazil. Amer J Public Health, 99(11), 1-11 and
SASCAT tool: De Silva and Harpham (2007). Maternal social capital and child nutritional status in developing countries. Health & Place, 13, 341-355.

Mother Study ID | | | | |

11. Maternal Depression Screener[8]

Read: Now I would like to ask you a few questions about you have been feeling since giving birth to (INFANT NAME). Specifically, I would like for you to now recall how you have been feeling IN THE PAST 2 WEEKS, not just how you feel today.

Instructions: Please read aloud the 4 answer choices before the respondent answers.

READ: Over the last 2 weeks, how often have you been bothered by any of the following problems:

11.1 ***Feeling down, depressed, or hopeless***	01 = Not at all<br>02 = Several days<br>03 = More than half of the days<br>04 = Nearly every day<br><br>05 = Other. Specify ___________________<br>88 = Don't Know	[ ][ ]
11.2 ***Little interest or pleasure in doing things***	01 = Not at all<br>02 = Several days<br>03 = More than half of the days<br>04 = Nearly every day<br><br>05 = Other. Specify ___________________<br>88 = Don't Know	[ ][ ]

[8] Patient Health Questionnaire-2 (PHQ-2)
Gjerdingen D, Crow S, McGovern P, Miner M, Center B. Postpartum depression screening at well-child visits: validity of a 2-question screen and the PHQ-9.Ann Fam Med. 2009;7(1):63-70.
Kroenke K, Spitzer RL, Williams JB. The Patient Health Questionnaire-2: validity of a two-item depression screener. Med Care. 2003;41(11):1284-92.

12. Women's Empowerment

Read: Now I would like to ask you a few questions about your household and your role in your household.

12.1	***During the last 6 months, who has been the decision maker on ….***			
	01 = Respondent (wife)<br>02 = Husband<br>03 = Mother-in-law<br>04 = Other wife of respondent's husband<br><br>05 = Other. Specify ________________<br>88 = Don't know<br><br>***Please record ALL responses.***	***12.1 A***	***…how household income is spent?***	
		12.1 B	***… your own health care?***	
		12.1 C	***… major household purchase (such as a cow, radio, TV, etc.)?***	
		12.1 D	***… purchasing of daily household needs (such as rice, oil, fuel)?***	
		12.1 E	***… children's health care?***	
		12.1 F	***… child's food and drinks?***	
		12.1 G	***… child's feeding problems?***	
		12.1 H	***… whether you attend support groups?***	

12.2	***Are you engaged with any savings program from your own income/ family income?***	01 = Yes<br>02 = No<br><br>88 = Don't know	
12.3	***Can/do you go to a hospital or health center or Immunization center alone or accompanied by your children for a health problem?***	01= Goes or can go alone to health center (Union sub-center, Community clinic, NGO clinic<br>02= Goes or can go alone to hospital (Upazilla Health Complex)<br>03= Goes or can go alone to immunization center<br>04= Goes or can go with children to health center(Union sub-center, Community clinic, NGO clinic)<br>05= Goes or can go with children to hospital(Upazilla Health Complex)<br>06= Goes or can go with children to immunization center<br>07=Cannot go to health center or hospital or immunization center alone or with children<br><br>88 = Don't know	

13. Maternal Program Participation & Handwashing

Read: Now I would like to ask you a few questions about a few other activities in your life.

13.1	***Have you ever received counseling for breastfeeding and/or complementary feeding related issues?***	01 = Yes<br>02 = No >> Skip to 11.3<br><br>88 = Don't know >> Skip to 11.3	
13.2	***How many times in the last 6 months?***	# of counseling sessions/visits<br><br>88 = Don't know	
13.3	***Have you participated in a mother-to-mother support group where you discussed breastfeeding and/or complementary feeding related issues?***	01 = Yes<br>02 = No >> Skip to 11.5<br><br>88 = Don't know >> Skip to 11.5	
13.4	***How many times in the last 6 months?***	# of support groups attended<br><br>88 = Don't know	
13.5	***Has your husband or the male head of household attended men's groups where they discussed breastfeeding and/or complementary feeding?***	01 = Yes<br>02 = No >> Skip to 11.5<br><br>88 = Don't know >> Skip to 11.5	
13.6	***How many times in the last 6 months?***	# of times attended<br><br>88 = Don't know	
13.7	***Have you attended EPI and growth monitoring sessions?***	01 = Yes<br>02 = No >> Skip to 11.9<br><br>88 = Don't know >> Skip to 11.9	
13.8	***How many times in the last 6 months?***	# of sessions attended<br><br>88 = Don't know	

Read: Now I would like to ask you a few questions about handwashing.

13.9	***In general, do you wash your hands with soap …***		
01 = Yes<br>02 = No<br><br>03 = Other. Specify ___________	***13.9 A***	*... Before preparing foods?*	
	13.9 B	*... Before eating foods?*	
	13.9 C	*... Before feeding (INFANT NAME)?*	
	13.9 D	*... After helping (INFANT NAME) go to the bathroom?*	
	13.9 E	*... After using the toilet?*	
13.10	***In general, does someone help (INFANT NAME) wash his/her hands with soap …***		
01 = Yes<br>02 = No<br><br>03 = Other. Specify ___________	***13.10 A***	*... Before eating foods?*	
	13.10 B	*... After he/she goes to the bathroom?*	

14. Anthropometrics

Read: Now I would like to take your height and weight measurements. Also, a finger prick blood sample (which requires only a small drop of blood) will be taken. This is a standard, regular way to quickly and safely check the amount of iron that is in your blood. Iron is an essential micronutrient, which is needed for important body functions for both young children and adults.

MEASUREMENTS FOR MOTHER		
14.1	HEIGHT IN CENTIMETERS	CM ___ ___ ___ . ___
14.2	WEIGHT IN KILOGRAMS	KG ___ ___ . ___
14.3	HEMOGLOBIN IN FINGERPRICK BLOOD SAMPLE	gm/dL ___ ___ ___ . ___
14.4	STATUS OF HEIGHT, WEIGHT, AND HEMOGLOBIN MEASUREMENTS<br>Measured …………… 1<br>Not Present………… 2<br>Refused all………… 3<br>Refused Hb test, weight OR height 4<br>Other ……………… 7	___

Read: Now I would now like to also take the length, weight, head circumference, and iron measurements of (INFANT NAME). Again, a finger prick blood sample (which requires only a small drop of blood) will be taken, in order to check the amount of iron that is in your baby.

ANTHROPOMETRIC MEASURES AND IRON STATUS FOR CHILD (9 MONTHS)		
14.5	RECORD NAME OF INFANT FROM 1.9	NAME ________________
14.6	RECORD SEX OF INFANT FROM 1.10	01 = Male<br>02 = Female ___ ___
14.7	WEIGHT IN KILOGRAMS	KG ___ ___ . ___
14.8	LENGTH IN CENTIMETERS<br><br>Instructions: Measurement should be taken when infant is lying down as flat as possible on his/her back.	CM ___ ___ ___ . ___
14.9	HEAD CIRCUMFERENCE IN CENTIMETERS	CM ___ ___ ___ . ___
14.10	HEMOGLOBIN IN FINGERPRICK BLOOD SAMPLE	gm/dL ___ ___ ___ . ___
14.11	STATUS OF HEIGHT, WEIGHT, HEAD CIRUMFERENCE, AND HEMOGLOBIN MEASUREMENTS<br>Measured ……... 1<br>Not Present…… 2<br>Refused all…… 3<br>Refused some test(s) 4<br>Other ………… 7	___

Mother Study ID | | | |

Was anyone other than the respondent present at the time of interview? | |

01=Yes
02=No

If 'Yes' record '01' Yes for each individual present.

Husband		Mother-in-law	
Father-in-law		Elder sister-in-law/Sister-in-law	
Uncle		Aunt	
Elder son / son		Daughter	
Mother (of Mother/Interviewee)		Father (of Mother/Interviewee)	

Other household member(s) | | *Please specify:*

Name: Relation:

Name: Relation:

Name: Relation:

Neighbor(s) | | *Please specify:*

Name: Relation:

Name: Relation:

Name: Relation:

Notes

Name of Interviewer ______________________ Code | | | | |

Name of Quality Controller ______________________ Code | | | | |
